# Supplementary material for: Endogenous learning for green hydrogen in a sector-coupled energy model for Europe
Source: Nat Commun. 2023 Jun 23;14:3743. doi: 10.1038/s41467-023-39397-2 (PMC10290158; doi:10.1038/s41467-023-39397-2)
Supplement: Supplementary file 1 — Supplementary Information [file 41467_2023_39397_MOESM1_ESM.pdf]

Supplementary Material

Endogenous learning for green hydrogen in a sector-coupled energy model for Europe

Elisabeth Zeyen[1,2*], Marta Victoria[3,4] and Tom Brown[1,2]

[1*]Department of Digital Transformation in Energy Systems, Faculty of Process Engineering, TU Berlin, Einsteinufer 25 (TA 8), Berlin, 10587, Berlin, Germany.
[2]Institute for Automation and Applied Informatics (IAI), Karlsruhe Institute of Technology (KIT), Forschungszentrum 449, Eggenstein-Leopoldshafen, 76344, Baden-Württemberg, Germany.
[3]Department of Mechanical and Production Engineering, Aarhus University, Inge Lehmanns Gade 10, Aarhus, 8000, Denmark.
[4]Novo Nordisk Foundation CO2 Research Center, Gustav Wieds Vej 10, Aarhus, 8000, Denmark.

*Corresponding author(s). E-mail(s): e.zeyen@tu-berlin.de;
Contributing authors: t.brown@tu-berlin.de; mvp@mpe.au.dk;

Contents

Supplementary method 1
Perfect foresight 1
Demand 2

More detailed discussion of the main results 3
Comparison to other studies 3

Limitations of this study 5

Further sensitivity analysis 6
Limit build out rates of renewable generation capacity 6
Costs and transition speed of the transport sector 8
Endogenous learning batteries and blue hydrogen production 10
Investment costs of electrolysis 14
Spatial resolution 18

Further analysis of main results 22
Investment cost and installed capacities of renewable generation 22
Composition of the investment costs 22
Investment cost and capacities of solar and wind in our main results 22
Annualised total system cost 25
Annualised total system cost difference between the three budgets . 25
Annualised total system cost difference between the methods 26
Energy balances 29
Electrolysis duration curve 39

Technology assumptions 39

Energy balances tables 44

Acronyms 56

Supplementary method

Perfect foresight

In this study, a time span of 2020-2050 with 7 investment periods of 5 years each ([2020,2025,...,2045,2050]) is considered and the total system costs are minimised. The modelling of these multiple investment periods is implemented in one single optimisation problem (perfect foresight) and allows a change in the installed capacities of the assets in each investment step. The investment costs of each asset are annualised and distributed over the years in which the asset is active (from build year until the end of the lifetime). This allows the costs in the individual investment periods to be weighted (e.g. with a social discount rate) and the costs of savings do not have to be taken into account at the end of the modelling horizon (which is the case if the total investment is considered instead of the net present value (NPV) in each time step).

The annualised investment costs c_s $[\frac{€}{MWa}]$ of an asset s are defined as

$$c_s = k_s \cdot \frac{i_s}{1 - \frac{1}{(1+i_s)^{L_s}}}. \tag{1}$$

k_s represent the specific investment cost $[\frac{€}{MW}]$, i_s the discount rate, and L_s the lifetime of the asset.

The annual costs of one single year a consists of fixed and operational costs. Fixed annualised investment costs of the power capacities G_s, energy capacities E_s and inter-connector capacities F_l are considered for all active assets (build year $b_s < a <$ end of lifetime $b_s + L_s$). Operational costs o_t are defined for power dispatch through generation and storage g_t, as wells as power flow through inter-connectors f_t at every time step t. Inter-connectors represent the conversion from one energy carrier to another (e.g. an electrolysis converting electricity to hydrogen). The total annual costs f_a is defined as

$$\begin{aligned} f_a = & \sum_{s|b_s \leq a \leq b_s + L_s} c_s \cdot G_s + \sum_{s|b_s \leq a \leq b_s + L_s} c_s \cdot E_s + \sum_{l|b_l \leq a \leq b_l + L_l} c_l \cdot F_l && \text{(fixed costs)} \\ & + \sum_{s|b_s \leq a \leq b_s + L_s, t} o_s \cdot g_{s,t} + \sum_{l|b_l \leq a \leq b_l + L_l, t} o_{l,t} \cdot f_{l,t}. && \text{(operational costs)} \end{aligned} \tag{2}$$

The total system costs for the whole investment period are minimised resulting in the objective function

$$\min \sum_a w_a f_a. \tag{3}$$

w_a represents a weighting of the annualised costs depending on the duration of the investment period and the social discount rate r. The annual cost of one year a then

discounted by

$$d_a = \frac{1}{(1+r)^{a_0-a}} \tag{4}$$

For example for the year a=2022, starting year a_0=2020 and a social discount rate of r=0.02

$$d_{2022} = \frac{1}{(1+0.02)^{(2022-2020)}} = 0.96. \tag{5}$$

The weighting w_a is the sum of all the years within the investment period, so e.g. for the period 2020-2025

$$w_{2020} = \sum_{a=2020}^{2025} d_a = 4.8. \tag{6}$$

In this study a social discount rate of $r = 0$ is assumed which results in an equal weighting of all investment periods of w_a = 5.

Demand

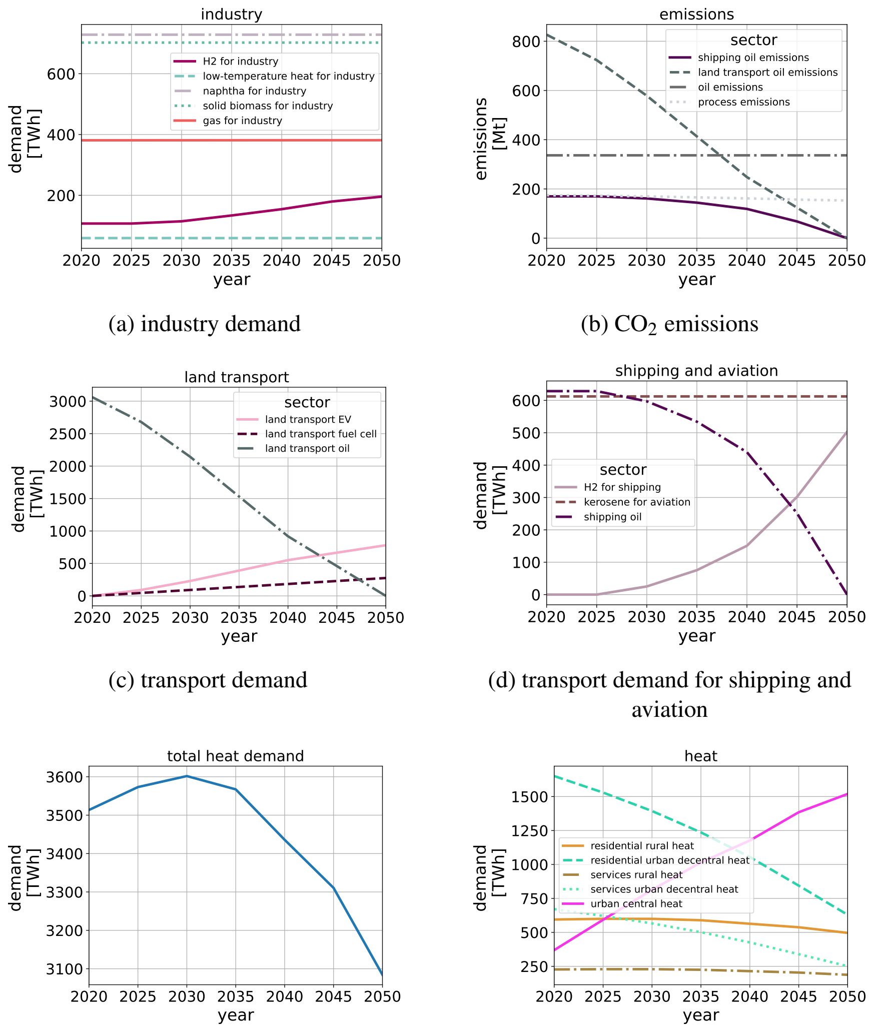

(a) industry demand

(b) CO_2 emissions

(c) transport demand

(d) transport demand for shipping and aviation

(e) heat demand

(f) heat demand by heating system

Fig. S1: Exogenous demand assumptions during the planing horizon for industry, transport and heating sector. As well as exogenous CO_2 emissions of industry processes and transport.

More detailed discussion of the main results

Comparison to other studies

In the following, we compare our findings concerning the cost of green hydrogen production to other studies. We have converted all cost assumptions into 2015-Euro (using the exchange rate of 1 USD = 0.876 Euro in 2020), assuming an annual

inflation rate of 2%. Our results of 1.32-1.99 €/kg$_{H_2}$ in 2030 with the endogenous method are in good agreement with studies by IRENA [1], Energy Transitions Commission (ETC) [2] and IEA [3] which all have production costs of green hydrogen below 2 €/kg$_{H_2}$ in 2030. Vartiainen et al. [4] find more favourable cost of green hydrogen production of 0.77-1.99 €/kg$_{H_2}$ in 2030. Compared to the optimised values in our study, they assume a higher number of full load hours of electrolysis and a larger capacity increase of solar and electrolysis. A JRC report [5] shows hydrogen production costs above our results in 2030 at 1.66-3.86 €/kg$_{H_2}$ due to slower capacity growth. In 2050, we find cost of green hydrogen production of 1.26-1.51 €/kg$_{H_2}$, which is in good agreement with results from ETC of 1.21 €/kg$_{H_2}$ [2], Hydrogen council of 1.4 €/kg$_{H_2}$ [6] and BloombergNEF of 0.68-1.55 €/kg$_{H_2}$ [7]. Vartiainen et al. [4] find lower costs 0.33-0.99 €/kg$_{H_2}$ due to greater capacity expansion. In contrast to our study, these studies assume an average electricity price and a fixed number of full load hours for electrolysis and thus cannot reflect the system advantage of electrolysis running at very low electricity prices. However, we only assume local learning for electrolysis and do not consider learning due to capacity expansion in other regions, while the other studies analyse global developments.

The electrolysis investment costs in our findings are below the projected investment costs in most other studies. For example, the highest investment costs in our scenarios with a +2°C budget are 380 €/kW$_{elec}$ in 2030, while Fraunhofer ISE finds 444 €/kW$_{elec}$ [8] and IEA 400 €/kW$_{elec}$ [9]. The costs for producing green hydrogen are similar compared to other studies since we have lower optimal full load hours. In 2050, we find investment costs of electrolysis between 75-95 €/kW$_{elec}$, which is well below the assumptions of IEA and IRENA of 200 €/kW$_{elec}$ [9,10]. Vartiainen et al. [4] find investment costs in the lower range of our findings of 80 €/kW$_{elec}$.

Odenweller et al. [11] show that fast scale-ups of electrolysis capacities until 2030 as we see in our +1.5°C scenarios may be infeasible. However, in this study we want to show the cost-optimal capacities that are necessary to achieve a given CO_2 budget. We do not consider hydrogen imports in this study. Imports would results in lower installed electrolysis and renewable capacities in Europe. Seck et al. find hydrogen imports of 15% [12], IRENA 17-50% [1] of total demand in 2050. The role of blue hydrogen differs between the studies as well. Studies by ETC and BloombergNEF [2,7] show that for most European countries, green hydrogen production is cheaper than blue hydrogen production, which is consistent with our results. Several studies [5,6,12] find a combination of both blue and green hydrogen production cost optimal. For example, Seck et al. [12] find a share of 20-52% of blue hydrogen in total production. In comparison to our study, Seck et al. [12] assume a seven times higher CO_2 storage potential and limit the installation pace of renewable capacities to historical levels, which makes blue hydrogen production more favourable. This is in agreement with our sensitivity analysis included in the Supplementary Material.

Our CO_2-unconstrained scenario is less expensive than scenarios with a CO_2 budget. The study by Way et al. [13] shows that a scenario that follows historical trends and

does not allow the transformation of comparatively expensive sectors is more costly than a scenario with a +1.5°C budget. However, unlike the scenario in [13], in the CO_2-unconstrained scenario parts of the energy system can transform if it is cost-effective.

Limitations of this study

The presented results contain several limitations, first in the way the experience curves are modelled, and second in the general scenario assumptions. We have prioritised the limitations based on our subjective view of their impact on the results.

First, a major impact on the results is the simplified assumption that hydrogen electrolysis costs are subject to local learning and renewable capacity costs are subject to global learning. If electrolysis are scaled up e.g. in the US and China, this accelerates technology learning and lowers the investment costs in Europe as well. Second, we assume no import of green hydrogen. This would lead to lower renewable and electrolysis capacities in Europe. However, globally, electrolysis would have to be built to satisfy the European demand, thus lowering investment costs. Third, the results are further limited by the exogenous assumptions in the transport sector which specify the share of ICE for a certain period and the share of hydrogen demand for shipping. These fixed shares have a large impact on the results, especially on scenarios with a +1.5°C budget. To stay within the given budget, hydrogen is required in 2020-2035 to produce synthetic fuels, while with a higher share of electric vehicles this would not be necessary. The impact of the transport transition pathways is analysed in the Supplementary Material. Further, the assumed current global cumulative capacity for the experience curves significantly influences the results. For example, if the global capacity of electrolysis is assumed to include the capacities of chlor-alkali electrolysis, the cost reductions through technology learning would be significantly lower. If, on the other hand, one assumes the capacities of PEM or SOEC, which are well below 1 GW today, the investment costs would decrease more. To keep the computational effort reasonable, no network infrastructure is included. Networks may have potential bottlenecks that affect the results. We have included in the Supplementary Material a sensitivity analysis concerning the spatial resolution for the exogenous method (see Figure S17a). A higher spatial resolution increases capacities of electrolysis, which result in lower investment costs of electrolysis for the endogenous and sequential method. The here presented results therefore provide an upper bound on investment costs for electrolysis and a lower bound on system costs with respect to the limitation of spatial resolution. We further only model technology learning for investment costs, but not for fixed operational and maintenance cost (FOM), efficiencies or lifetimes, which are assumed exogenously based on the year of construction and the respective technology. In particular, an improvement in the efficiency of electrolysis could lead to lower capacities or increased use of hydrogen. No spill-over effects between technologies are taken into account. These effects could be important for example for on- and offshore wind, but also for spill-over between different electrolysis types. We assume all plants are large (>100 MW)

and disregard the effects of scaling the size of individual electrolysers. In addition, we do not limit the annual maximum expansion of electrolysis capacity. If this condition would be binding, it would lead to a greater use of grey or blue hydrogen and higher investment costs for electrolysis. We model a fixed weighted average cost of capital (WACC) of 7%, but do not model country-dependence or learning in financing that would decrease the WACC as banks and investors become more comfortable with a new technology[14]. Additional benefits of a faster reduction of CO_2 emissions, such as those related to new jobs or health are not considered within the optimisation. Taking these factors into account could significantly increase the costs of the +2.0°C compared to the +1.5°C scenarios. We do not investigate the impact of different social discount rates. This is analysed in a previous publication[15]. Although we investigate the robustness of the results by varying learning rates, we do not consider the change of a learning rate during a scenario, e.g. the learning rate of hydrogen electrolysis could be 16% between 2020-2035 and decrease to 10% thereafter. Finally, we consider only one factor experience curves and not, for example, learning-by-research as an additional factor.

In this paper, we have classified all hydrogen produced by electrolysis as green, even though conventional power plants are still part of the generation mix, especially up to 2035. In all scenarios, the renewable generation is greater than the electricity demand of the electrolysis at every modelled hour. It is therefore possible, that all hydrogen produced via electrolysis is green, even though conventional power plants remain contributing to the generation.

Future work should investigate limitations with potentially large impacts such as imports of hydrogen, full endogenisation of the transport sector and higher spatial resolution.

Further sensitivity analysis

Limit build out rates of renewable generation capacity

The high expansion rates of renewable energies are challenging as new plants often have long planning phases, are delayed or the construction is stopped by lawsuits or by environmental protection reasons. In the following, we therefore examine scenarios in which the expansion rates of onshore and offshore wind as well as solar PV are limited respectively to 31 GW, 13 GW and 52 GW annual new capacity additions in Europe. These expansion rates are derived from the maximum historical expansion rates in Germany (4.9 GW in 2017, 2.1 GW in 2015, 8.2 GW in 2012[16]), which are scaled up for Europe in proportion to the population. The expansion of renewables is not constrained in the main results, as this often predetermines the transition paths and larger expansion rates than the historical ones are possible per se.

The installed capacities are significantly lower for solar PV and onshore wind with limited expansion rates (see figure S3). Offshore installations are increasing to

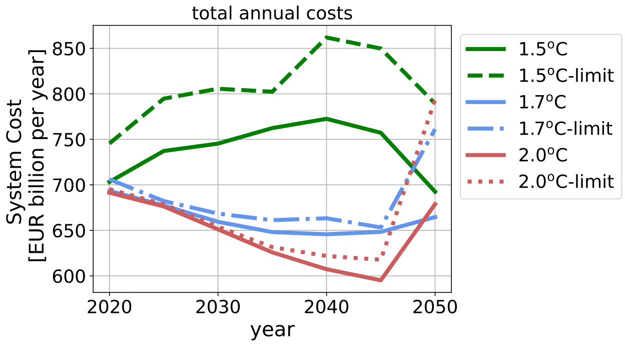

Fig. S2: Comparing total annual system costs for scenarios with and without renewable build out rates.

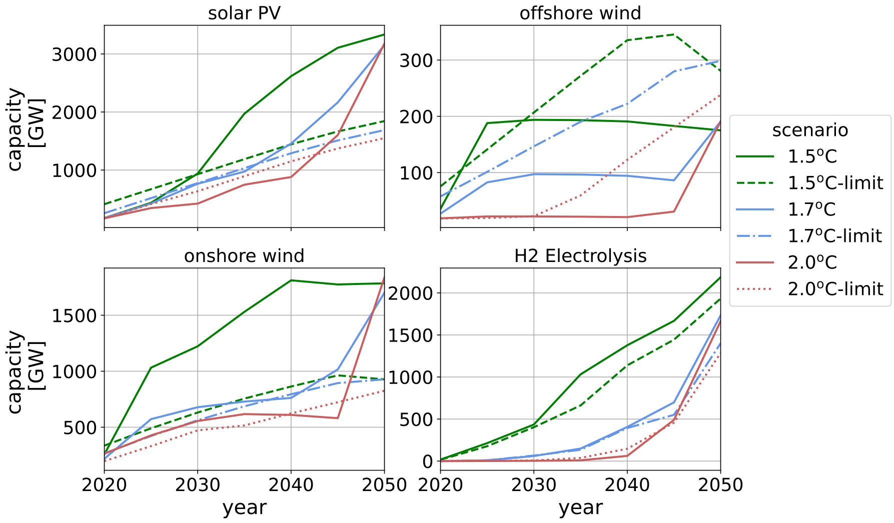

Fig. S3: Installed capacities of renewable generation and electrolysis for different CO_2 budgets assuming global learning for renewable generation and limited growth rates.

compensate for the low capacities of solar PV and onshore wind. The total costs are about 4-9% higher than in the non-limited scenarios, depending on the budget (see figure S2). Less hydrogen is produced and used since the electricity prices are higher. Nuclear power is used since not all of the electricity demand can be covered by renewables due to the growth limit (see figure S4). This causes higher system costs.

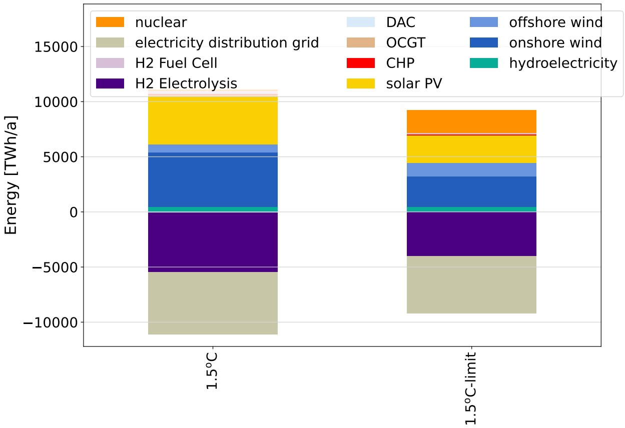

(a) Electricity

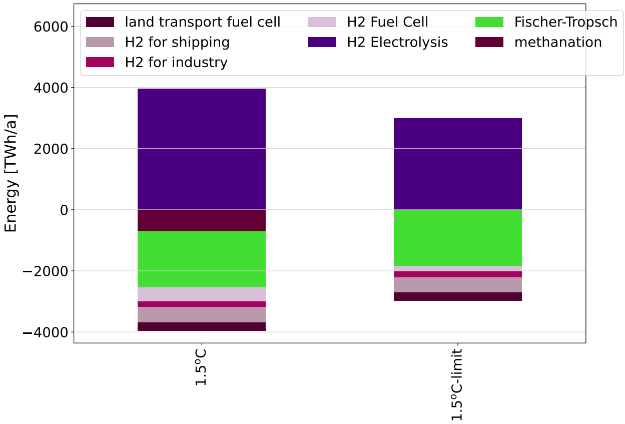

(b) hydrogen

Fig. S4: Comparing electricity and hydrogen supply and demand for scenarios with a +1.5°C budget with and without growth limit of renewables.

Costs and transition speed of the transport sector

The proportions of internal combustion engine, electric and fuel cell cars are exogenously specified in this study. In this sensitivity analysis, on the one hand, the additional costs of cars and charging infrastructure are estimated, and on the other hand, the effects of a slower transformation of land transport are illuminated.

Vehicles are divided into (i) light duty vehicles (passenger cars, freight transport, 2-wheelers) and (ii) heavy duty vehicles (buses, motor coaches, trolleys, heavy duty freight). Total number of vehicles and demand shares are taken from the JRC-IDEES 2015 [17] and considered fixed throughout the transition. The cost and lifetime

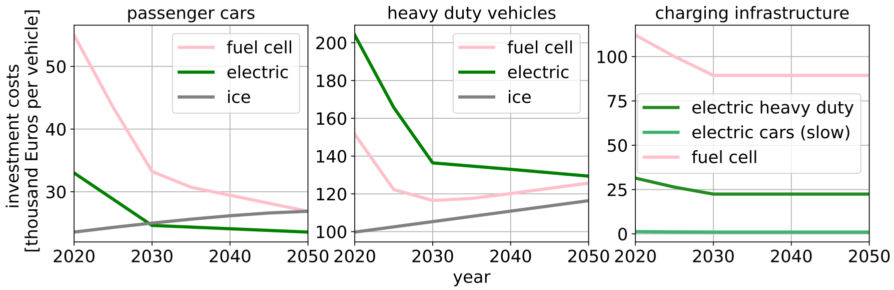

Fig. S5: Investment costs for passenger and heavy duty vehicles depending on engine type (electric vehicles (*electric*), internal combustion engine (*ice*) and fuel cell (*fuel cell*) cars), as well as investment costs for charging infrastructure. No infrastructure costs for current internal combustion engines are assumed.

assumptions for passenger cars, heavy duty vehicles and charging infrastructure are taken from the Fraunhofer ISE study[18] (see Figure S5). The investment costs are annualised assuming a discount rate of 7%. For the passenger cars one charger per car is assumed, for the heavy duty vehicles fast charging with 20 vehicles per charger is assumed. This is a rather conservative assumption, e.g. compared to the Bloomberg report[19] which assumes a rise from today's 5-20 vehicles per charger to 30-40 vehicles per charger by 2050. No costs are assumed for the existing infrastructure for internal combustion vehicles. The resulting annualised costs for cars and charger infrastructure for the different transport scenarios are added after the optimisation to the annualised system costs.

In our base scenarios we consider a fast change in the transport sector. This assumes that by 2040, all vehicles are completely electrified or hydrogen-powered. In order to shed light on the impacts of a slower transformation of the land transport sector, two further transformation paths are highlighted in this sensitivity analysis with a medium and slow transformation of the sector. For all three budgets the fast transition of the land transport sector is cost-optimal. The assumption of a fast change in the transport sector, which is stated in the main results, is therefore justified for all three budgets. If costs for vehicles and charging infrastructure are included, total costs are between 1-2% higher with a slower transformation from the +2.0°C to the +1.5°C budget (see Figure S7). The costs for cars and charging infrastructure make up a large part of the total costs with a share of 59-67%. The proportionally small cost difference of 1-2% of total costs can be explained by the large share of transport sector costs. However, a faster transition of the transport sector leads to savings of 538-1722 billion euros over the entire planning horizon. With a faster transition of the land transport sectors, electrolysis capacities are built up earlier. In 2050, investment costs and installed capacities of electrolysis are comparable for different speeds of the transport sector.

The investment costs for electrolysis in 2050 are similar between the scenarios. Our

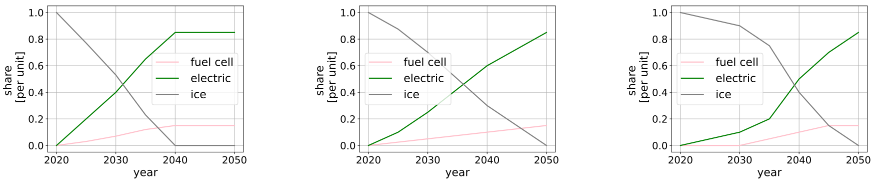

(a) fast (b) medium (c) slow

Fig. S6: Shares of electric, fuel cell and internal combustion engine (*ice*) for the different speeds of transformation of the transport sector. In the main result section, the fast transition of the transport sector is assumed.

results for the investment costs of hydrogen electrolysis should therefore be robust for 2050, even in the case of a slower transformation of the land transport sector. In 2030, investment costs of electrolysis is even lower in the scenario with a slower transformation of the transport sector, as the demand for hydrogen is higher. The higher demand for hydrogen is driven by the larger demand for fuel for internal combustion engine vehicles, which has to be partly met by synthetic fuels in order to stay within climate targets.

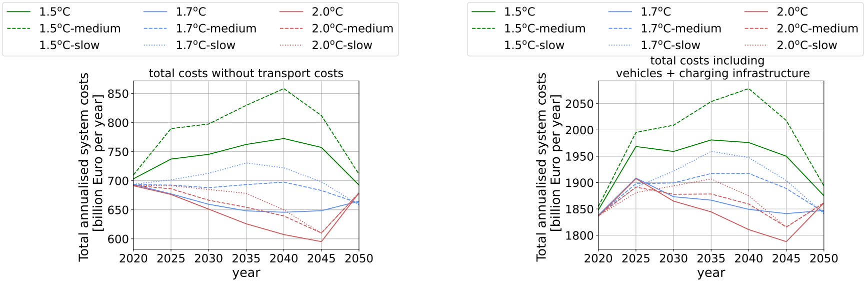

(a) annualised system cost without costs for vehicles and infrastructure

(b) annualised system cost including costs for vehicles and infrastructure cost

Fig. S7: Total annualised costs for three different transition speeds of the transport sector and without and including costs for charging infrastructure and cars. The slow transport scenario with a +1.5°C budget is not feasible and therefore not displayed in the graphics.

Endogenous learning batteries and blue hydrogen production

The choice on which technologies endogenous learning is applied impacts the results. Two further scenarios are therefore considered as a sensitivity analysis. First, to better represent the competition between blue and green hydrogen production, a scenario with endogenous learning is assumed for SMR with carbon

capture (LR_{SMR}=10%, capture rate 90%) and no learning on electrolysis and renewable. Second, endogenous learning is assumed for batteries ($LR_{battery, energy}$=18%, $LR_{battery, power}$=19%) in combination with endogenous learning of solar PV. In both scenarios, endogenous learning does not lead to increased use of either technology (SMR + CC or batteries).

To investigate the conditions under which blue hydrogen production is used, we perform a sensitivity analysis on three key parameters for blue hydrogen production (i) the investment cost, (ii) the CO_2 sequestration potential and the (iii) the capture rate. The following results are performed with the exogenous method and a CO_2 budget of +1.7°C.

Investment costs

We vary the investment costs between 0 to 572 Eur/kW_{H2} (our base cost assumption). For CO_2 storage potential and carbon capture rate we assume our base assumptions of 200 Mt_{CO_2} per year and 90% respectively. The option of blue hydrogen is used if the investment costs are below 286 EUR/kW_{H2}. However, most of the hydrogen is produced via green hydrogen, even if blue hydrogen production is free of charge (see Figure S8). There are three reasons for this. First, electrolysis offers the system advantage in the way that electricity from renewable sources can be used, which would otherwise be curtailed. Second, blue hydrogen production does not capture all CO_2. Therefore, additional DAC or negative emissions from biomass with carbon and capture are needed to reach net zero emissions in 2050. Third, the limited CO_2 storage potential, which is always a binding condition in our scenarios.

CO_2 sequestration potential

We vary the CO_2 storage potential per year between 200 (base assumption) and 2000 Mt_{CO_2}/a. It is unclear how large the CO_2 storage potential is in Europe. Estimates vary between 108-7147 GtCO2[20]. We have assumed a sequestration potential of 200 Mt/a as a base value to account for uncertainties about CO_2 infrastructure and storage potential. Base assumptions for investment costs and carbon capture rate are assumed. As the storage potential increases, the amount of synthetic fuels produced and thus the amount of hydrogen production is reduced. Hydrogen is still mainly produced via electrolysis in 2050 (see Figure S9). Above a storage potential of 800 Mt_{CO_2}/a, about a small amount of 18 TWh_{H_2} (1% of total production) grey hydrogen is still produced in 2050. From a storage potential of 2000 Mt_{CO_2}/a, blue hydrogen is produced. In this case, the share of blue hydrogen in total production is 19%. It should be noted that with an annual sequestration of 2000 Mt_{CO_2} the CO_2 storage potential is exploited after 54 years based on a conservative assumption of a total storage potential of 108 Gt_{CO_2}.

Carbon capture rate

There is a wide range of assumed possible carbon capture rates of blue hydrogen production ranging from 53-99%, with higher rates being achieved primarily with autothermal reforming. In the following, we compare our base assumption of 90%

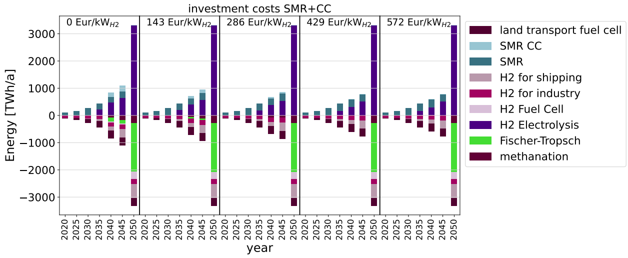

Fig. S8: Varying the investment costs of blue hydrogen production from 0 to 572 €/kW$_{H_2}$ (our base cost assumptions). CO_2 sequestration potential and capture rate are set at base assumptions (200 Mt_{CO_2} per year, and 90% respectively).

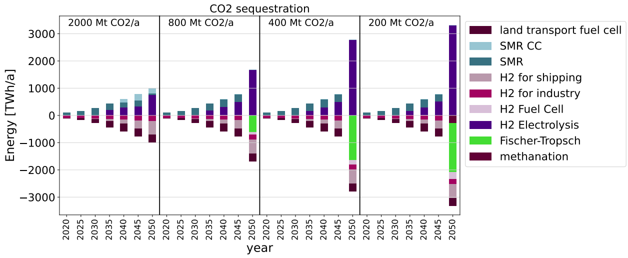

Fig. S9: Varying CO_2 sequestration potential per year from 200-2000 Mt_{CO_2} per year. SMR investment costs and capture rate are set at base assumptions (572 €/kW$_{H_2}$ and 90% respectively).

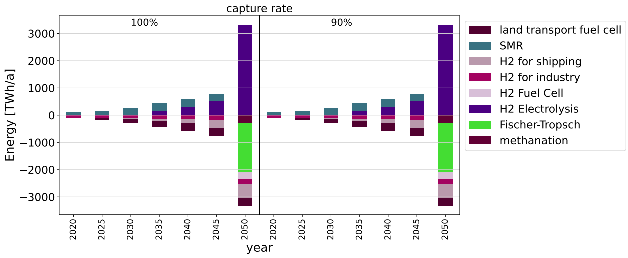

Fig. S10: Comparing carbon capture rate blue hydrogen production from 100% to our base assumptions of 90%. CO_2 sequestration potential and SMR investment costs are set at base assumptions (200 Mt_{CO_2}/a, and 572 €/kW$_{H_2}$ respectively).

capture rate with an assumed capture rate of 100%. In our scenarios, varying only the carbon capture rate has no impact on the results (see Figure S10).

Combined impact of two parameters on the results

In the following, we make the most optimistic assumptions for two parameters and vary the third. First, we consider scenarios in which we vary the CO_2 sequestration

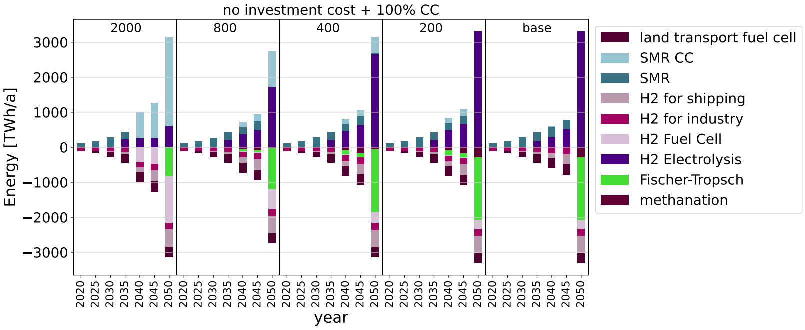

Fig. S11: Varying the CO_2 sequestration potential from 200-2000 Mt_{CO_2}/a with 100% carbon capture rate and no investment costs for blue hydrogen production. This is compared to our base scenario (right panel) with investment costs of blue hydrogen production of 572 €/kW_{H_2}, CO_2 sequestration potential of 200 Mt_{CO_2}/a, 90% carbon capture rate.

potential with no investment costs for the production of blue hydrogen and a carbon capture rate of 100%. With a storage potential of 200 Mt_{CO_2}/a, blue hydrogen is used under these assumptions in the years 2040-2045 with a maximum share of hydrogen production of 17% (see Figure S11). With increasing storage potential, the share of blue hydrogen production also increases to a share of 80% in 2050 at a storage potential of 2000 Mt_{CO_2}/a. A larger part of the hydrogen is converted back into electricity in fuel cells (1343 TWh_{H_2} in 2050) in this scenario. The CO_2 storage potential is fully utilised. With a sequestration potential greater than 2000 Mt_{CO_2}/a, hydrogen production would be completely switched to blue hydrogen.

In a second step, we consider a high sequestration potential (2000 Mt_{CO_2}/a) and a high carbon capture rate (100%) and vary the assumptions on the investment costs of blue hydrogen production. With our base cost assumptions of 572 €/kW_{H_2} 23% of the hydrogen is produced as blue hydrogen in 2050 (see Figure S12). The share is increasing with decreasing investment costs. With investment costs of 286 €/kW_{H_2} the share of blue hydrogen production is 49%.

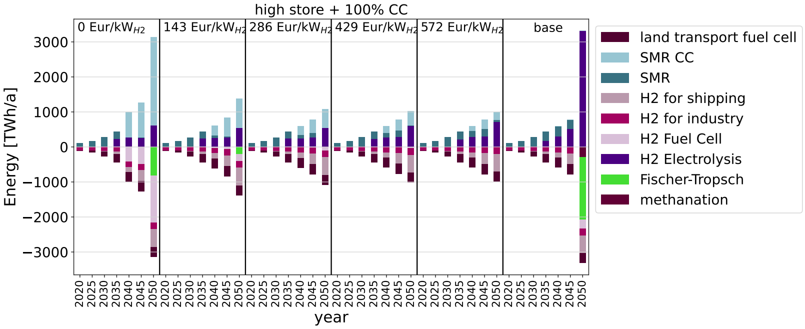

Fig. S12: Varying the investment costs of blue hydrogen production assuming high CO_2 sequestration potential of 2000 Mt_{CO_2}/a and 100% carbon capture rate. This is compared to our base scenario (right panel) with investment costs of blue hydrogen production of 572 €/kW_{H_2}, CO_2 sequestration potential of 200 Mt_{CO_2}/a, 90% carbon capture rate of blue hydrogen production.

Investment costs of electrolysis

The technology assumptions of electrolysis are taken from the Danish Energy Agency (DEA)[21] for a 100 MW alkaline electrolysis (AEC) plant. These costs, also called engineering, procurement and construction (EPC) price, consist of the equipment and installation costs. They include the expenses for stack, power electronics, gas conditioning, balancing of the plant and labour. The assumptions are for an electrolysis which produces hydrogen with a pressure of 35 bar and an additional waste heat steam of 50°C from electricity (400 V_{AC}) and purified water. The annual operational and maintenance costs are estimated in[21] based on current projects to be 2% of the investment costs. The cost of replacing the stack is not included in the fixed operational and maintenance cost (FOM) since it is assumed that the stack does not need to be replaced within the technical lifetime (lifetime of AEC stack is assumed to be more than 100 000 hours, electrolysis with 4000 full load hours and a lifetime between 25-35 years). The investment cost do not include costs for water purification, transformer costs or connection fees to the transmission system operators.

We are taken most of our technology assumptions of the DEA's technology catalogue since firstly it contains a large number of technologies and thus avoids picking particularly optimistic or particularly pessimistic assumptions for individual technologies, and secondly, this data is constantly updated. There are currently a few electrolysis units with capacities above 100 MW that are already operational and several projects are expected to be connected to the grid in the next two years[22,23].

In the following scenarios, we vary the assumptions about the initial investment costs firstly to reflect uncertainties regarding these (see Figure S13 for cost assumptions from different sources) and secondly to represent other types of electrolysis such as polymer electrolyte membrane (PEM) and solid oxide electrolyser cells (SOEC) with current higher investment costs (see Figure S14). We assume the same learning

rate of 18% for all following scenarios. The learning rates of PEM and SOEC are subject to higher uncertainty, as these technologies are not yet as mature as AEC. The learning rates for PEM vary between 13-30% [10,21,24,25] and for SOEC between 0-44% [26–28]. Higher learning rates than those assumed here result in a steeper learning curve and would thus reduce the costs for PEM and SOEC more quickly. This effect is not analysed in the following sensitivity analysis. The effects of unit-scaling also differ between the electrolysis types. SOEC investment costs depend more on the system size compared to AEC or PEM [29]. This is due to the fact that a smaller share of the total costs depends on the stack, which has a low cost impact on the system size due to its modularity. We use the endogenous method to represent the learning an scenarios corresponding to a +1.7°C budget. Our base initial investment cost c_0 of 650 Eur/kW$_{el}$ for AEC in 2020 according to DEA are increased by factors of 1.2 to 9 times.

Electrolysis is installed even with very high investment costs of 5850 €/kW$_{elec}$ and the investment costs decrease in all scenarios over the modelling horizon (see Figure S15). Initial costs of 780-1300 €/kW$_{elec}$, corresponding for example to uncertainties in cost assumptions of AEC or low to medium cost estimates for PEM, lead to investment costs of electrolysis in the range of 114-189 €/kW$_{elec}$ in 2050 and comparable volumes of hydrogen demand and supply (see Figure S16). Cost assumptions in the range of 1950-2600 €/kW$_{elec}$, corresponding to high cost assumptions of PEM or low cost estimates for SOEC, lead to investment costs of 284-379 €/kW$_{elec}$ in 2050 and increased use of grey hydrogen in the period 2020-2045. High investment costs of 5850 €/kW$_{elec}$ (corresponding to the highest cost assumptions in the datasets we discussed for SOEC), decrease hydrogen production by 15% in 2050 and 73% of the hydrogen is produced via grey hydrogen in 2045. Investment cost of electrolysis are 1257 €/kW$_{elec}$ in 2050.

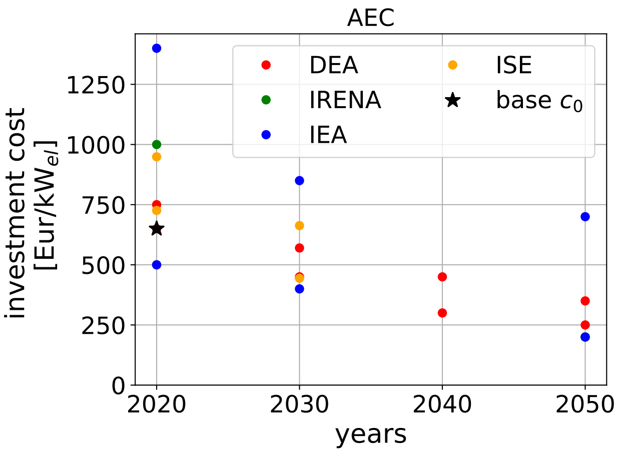

Fig. S13: Investment costs of alkaline electrolysis (AEC) from Danish Energy Agency (DEA)[21], International Renewable Energy Agency (IRENA)[10], International Energy Agency (IEA)[9], and Fraunhofer Institute for Solar Energy Systems (Fraunhofer ISE)[8]. If the source contains small and large system sizes both values are shown. Our base initial cost assumptions $c_0 = 650$ €/kW$_{\text{elec}}$ are from DEA for a 100 MW plant in the year 2020.

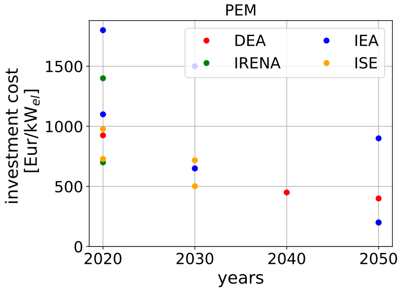

(a) PEM

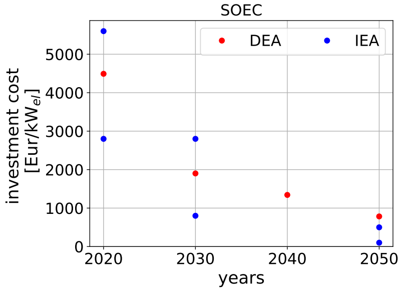

(b) SOEC

Fig. S14: Investment costs of PEM and SOEC from Danish Energy Agency (DEA)[21], International Renewable Energy Agency (IRENA)[10], International Energy Agency (IEA)[9], and Fraunhofer Institute for Solar Energy Systems (Fraunhofer ISE)[8].

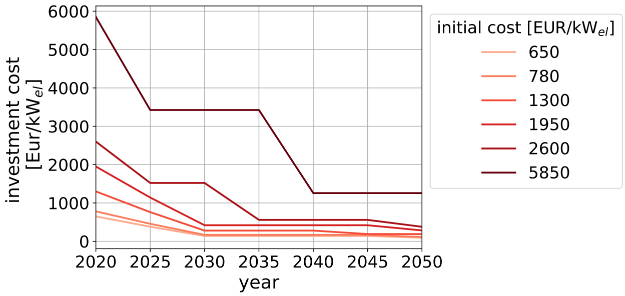

Fig. S15: Investment costs for the endogenous method with base learning rate of 16% and varying initial cost assumptions for scenarios with a +1.7°C budget.

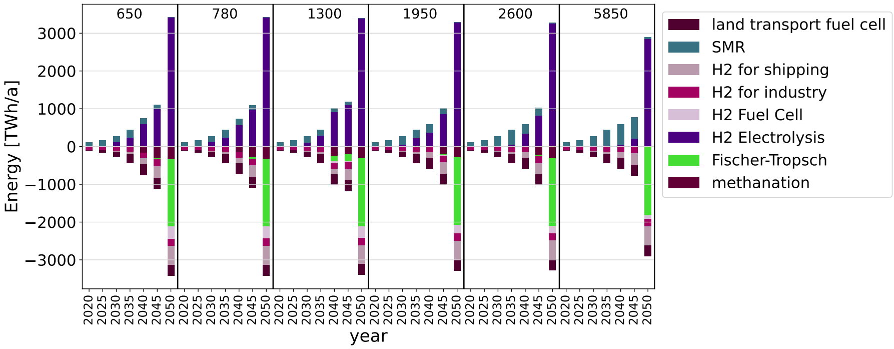

Fig. S16: Hydrogen balance for different initial investment costs (given in €/W_{elec} in the plot) of electrolysis.

Spatial resolution

In order to reduce the computational complexity with endogenous learning as a mixed integer problem (MIP), no grid infrastructure is represented in the main results and the renewable generation is modelled in six typical regions. This simplification can lead, on the one hand, to potential grid bottlenecks not being represented, and on the other hand to an underestimation of renewable generation by clustering different capacity factors of various regions[30]. In order to examine the impact of these assumptions on our results, scenarios with a higher spatial resolution of 37 regions are compared with the presented main scenarios of one region for the exogenous method. Current capacities for the electricity grid are assumed, which can be further expanded. The trade-offs between electricity and hydrogen grid are examined in more detail and with a higher spatial resolution in another publication by Neumann et al.[31].

With a higher spatial resolution, the capacities of electrolysis increase by 9-27% in 2050 from the tight +1.5°C to the +2.0°C budget and the production of hydrogen rises by up to 2% in 2050 compared to the scenarios without grid infrastructure (see Figures S17a, S18). The larger volume of hydrogen is used for re-electrification in fuel cells (see Figure S17b). The endogenous or sequential method in which investment costs depend or are adjusted based on the installed capacity would result in lower investment costs of electrolysis at higher spatial resolution.

Overall the electricity produced by renewables generation in 2050 reduces by 2-3% from the +2.0°C to the +1.5°C scenario with a higher spatial resolution since grid bottlenecks limit the distribution of renewable generation. The combination of different capacity factors results in lower generation of solar PV and onshore wind generation in the scenario without grid infrastructure and a higher generation of offshore wind. Larger renewable generation capacities are built with a higher spatial resolution. Solar PV, onshore and offshore wind capacities increase with a higher spatial resolution by 1-12%, 19-30% and 61-83% respectively in 2050 (see Figures S19, S20). More electricity is fed into the grid by nuclear power plants in 2050 (278-558 TWh with high spatial resolution compared to 115-120 TWh with low spatial resolution). This change in generation mix leads to lower hydrogen storage capacities with a higher spatial resolution (see Figure S21).

Total system cost over the whole modelling horizon increase by 12-16% from the +2.0°C to the +1.5°C scenarios with a higher spatial resolution (see Figure S22). The higher costs are caused primarily by higher costs for nuclear, renewable energies and costs for the electricity distribution and transmission network. Costs for the electricity and hydrogen grid contribute with a share of 0.1-7.6% to the annualised total costs. The largest costs are in 2050 with a share of 7.6% of the total costs for the +1.5°C budget. In the +1.5°C scenario cost of about 12 billion Euros per year account for the hydrogen network, 61 billion Euros per year for the electricity transmission grid.

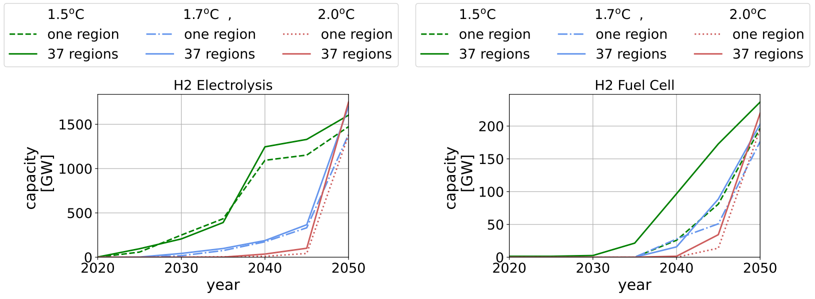

(a) Electrolysis (b) Fuel cell

Fig. S17: Installed capacities of electrolysis and fuel cells for the exogenous method, base learning rate assumptions, comparing scenarios without any modelled grid infrastructure with scenarios with a spatial resolution of 37 regions.

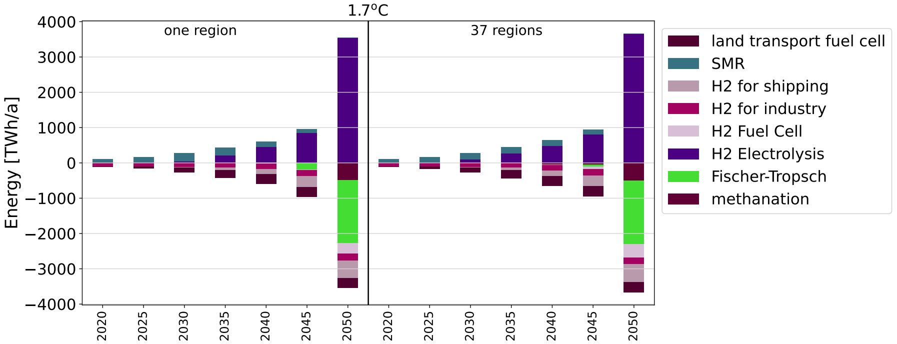

Fig. S18: Energy balance hydrogen for a +1.7°C scenario, comparing a scenario without any modelled grid infrastructure with a scenario with a spatial resolution of 37 regions.

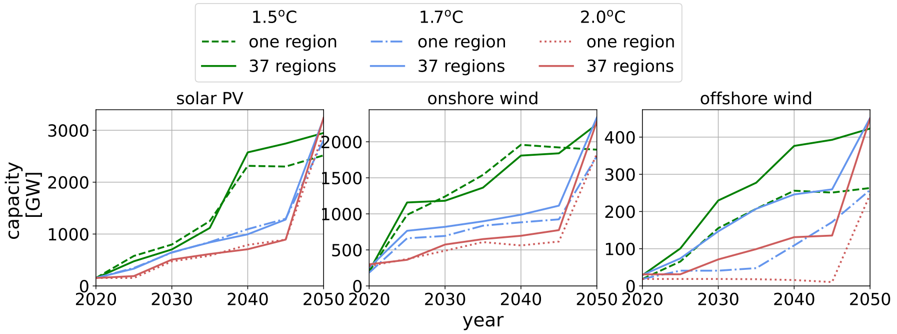

Fig. S19: Installed capacities of renewable generation for the exogenous method, base learning rate assumptions, comparing scenarios without any modelled grid infrastructure with scenarios with a spatial resolution of 37 regions.

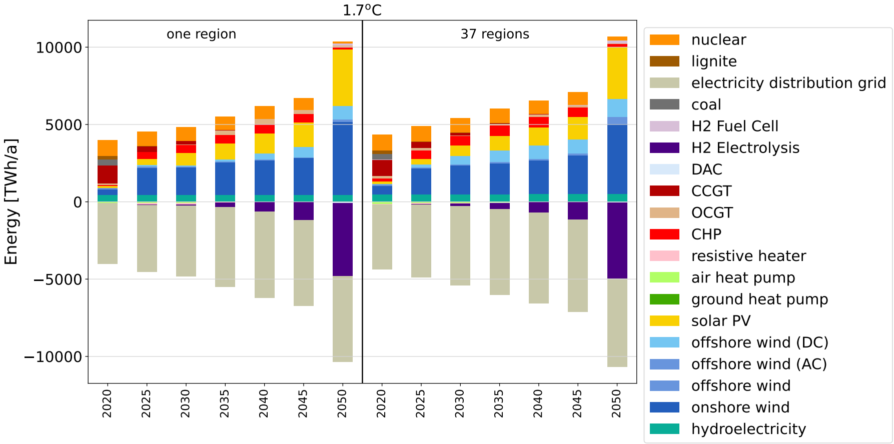

Fig. S20: Energy balance electricity (transmission level) of the +1.7°C scenario, comparing a scenario without any modelled grid infrastructure with a scenario with a spatial resolution of 37 regions.

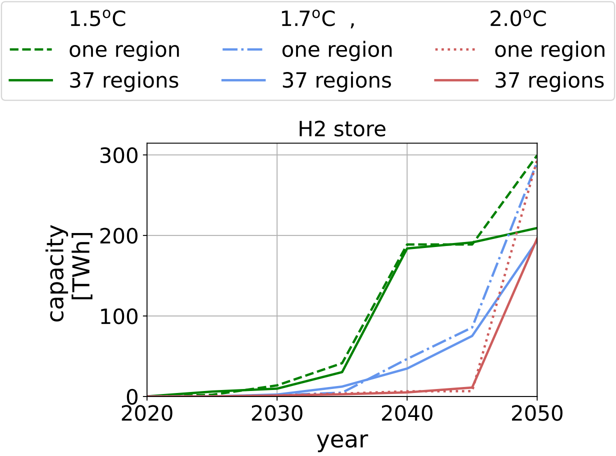

Fig. S21: Installed capacities of hydrogen storage for the exogenous method, base learning rate assumptions, comparing scenarios without any modelled grid infrastructure with scenarios with a spatial resolution of 37 regions.

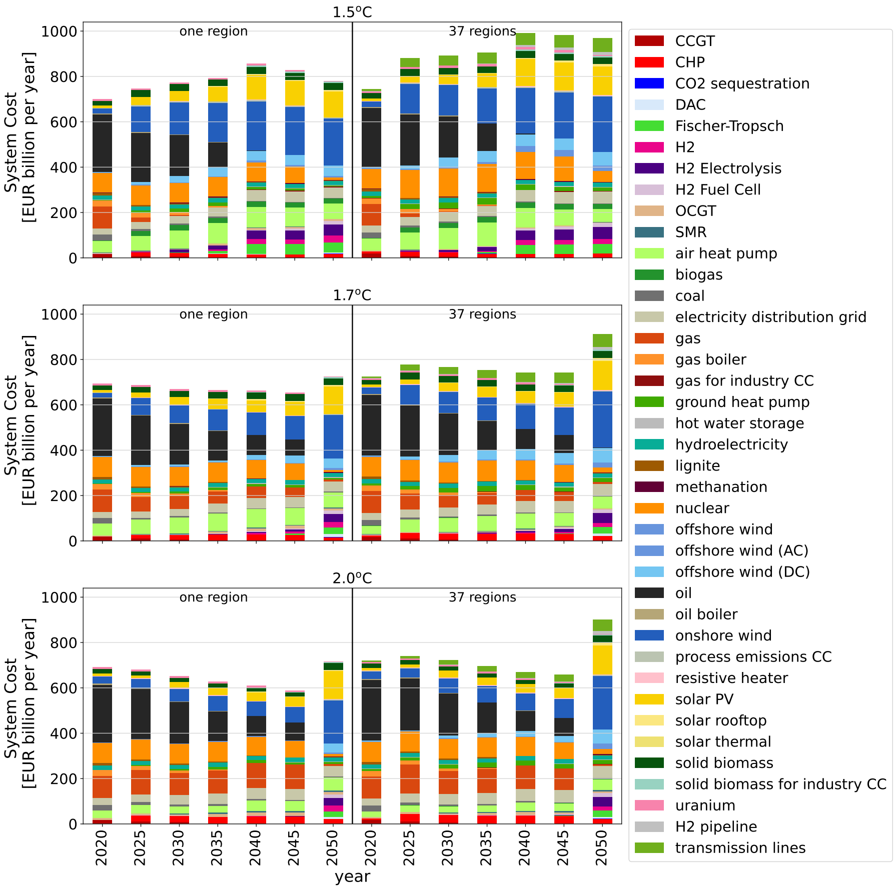

Fig. S22: Annualised system costs for the three different budgets, comparing a scenario without any modelled grid infrastructure with a scenario with a spatial resolution of 37 regions.

Further analysis of main results

Investment cost and installed capacities of renewable generation

Composition of the investment costs

In the following, we provide an overview of the composition of the investment costs for wind and solar. The exogenous cost assumptions are from the Danish Energy Agency technology data DEA[21] which also provides a detailed description of each technology. The technology assumptions depend on the build year of the respective asset. External grid connection costs are added to these investment costs in the model that do not undergo any learning. For offshore wind grid connection costs depend on the location and connection type (AC or DC), for solar and onshore wind additional grid connection costs of 133 €/kW are added.

The investment costs of onshore wind consist of the costs for equipment (turbine, foundation, cables), installation and development, cost of land, internal grid connection, decommissioning cost of existing turbines and other costs (for example compensation of neighbours living close to the wind park). The investment cost of offshore wind include cost for equipment (turbine, foundation, cables, grid connection), installation, project development and other costs (e.g. insurances, sea right fees, contingencies). Operating and Maintenance cost of wind farms include insurance, service agreement, repairs not covered by service agreement, land rent and administration. Solar PV investment costs include costs for the equipment (PV module, inverter, transformer, grid connection, balance of the plant), the installation and other costs (e.g. costs for permits, surveys, studies, planning, legal expenditures). Operational and maintenance cost of solar PV include insurance, land rent, cleaning of the modules, asset management and grass cutting.

Investment cost and capacities of solar and wind in our main results

The investment costs for renewable generation depend on the installed capacities in the sequential and endogenous method. For both methods, the resulting investment costs for all three carriers (solar PV, on- and offsore wind) are below the exogenous cost assumptions of DEA if a stricter budget of +1.5°C to +1.7°C is assumed. This is caused since larger capacities are built than assumed for the exogenous cost projections. The actual course of cost reduction differs between the endogenous and sequential method. With the endogenous method, investment costs decrease in earlier investment periods compared to the sequential method (see Figure S23) since capacities are scaled up faster (see Figure S24). This is because the sequential method does not have the foresight of how far costs can decrease, but only updates the assumptions on investment costs after optimisation, depending on the installed capacities. In contrast, the endogenous method sees the potential cost reduction and decrease in overall costs through a rapid expansion of renewable capacities. In addition, the technology mix differs between the methods. While the endogenous method expands solar PV more due to the anticipation of higher cost reductions from a higher learning rate, the sequential method expands more offshore wind. The exogenous method

generally leads to lower renewable capacities compared to the other two methods, as the investment costs are higher.

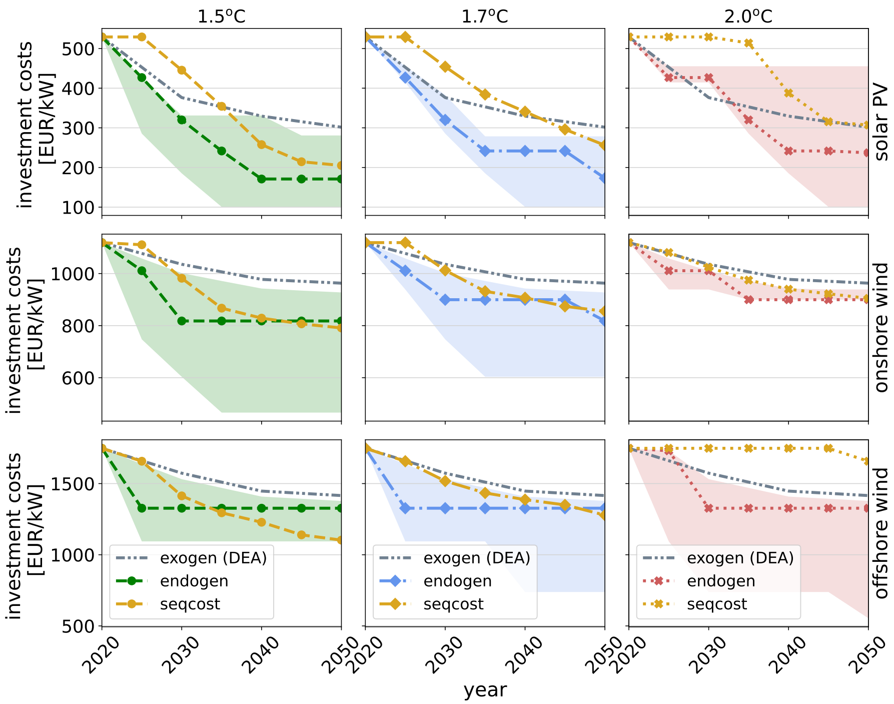

Fig. S23: Investment costs for renewable generation (*rows*) for different carbon budgets (*columns*) and the three different methods (i) exogenous (*exogen*), (ii) sequential cost (*seqcost*) and (iii) endogenous (*endogen*). Contour area indicates scenarios with ±10% variation of the learning rate for all technologies with endogenous learning. These investment costs are without grid connection costs which are added. For them no learning is assumed.

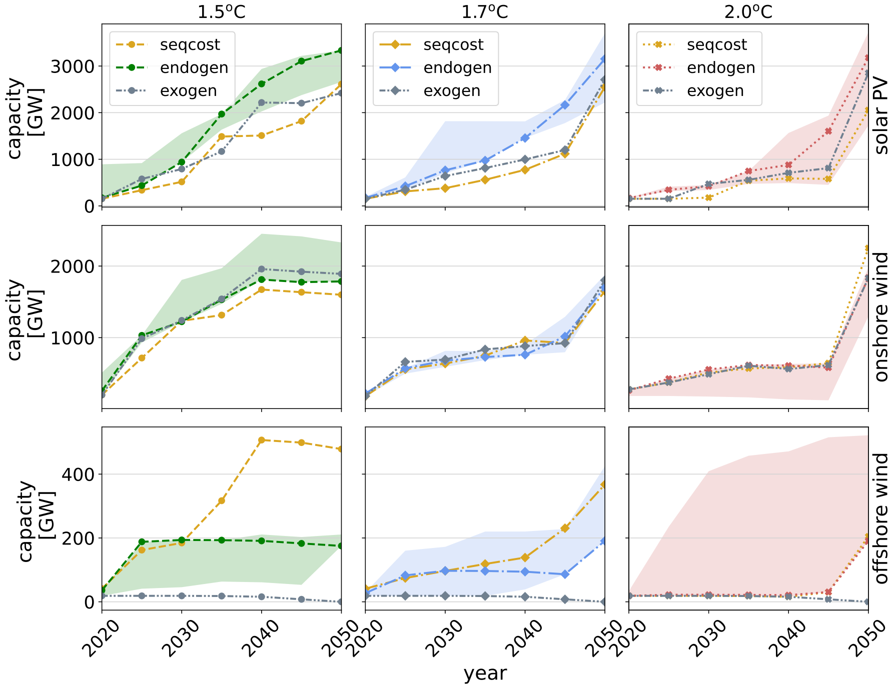

Fig. S24: Installed capacities of renewable generation (*rows*) for different CO_2 budgets (*columns*) and the three different methods (i) exogenous (*exogen*), (ii) sequential cost (*seqcost*) and (iii) endogenous (*endogen*). Contour area indicates scenarios with $\pm 10\%$ variation of the learning rate for all technologies with endogenous learning. Trade off between offshore and onshore wind depending on the learning rate which results in a large contour area when varying learning rate. Values are also shown in Tables S35, S36, S37.

Annualised total system cost

Total annualised system costs without estimated costs of climate change damage for the three different budgets and three different methods are shown in Figure S25. The +1.5°C budget leads to higher annualised costs compared to the +1.7°C and +2.°C scenarios if estimated costs of climate change damage are not included. However, in 2050 the total annualised costs only vary between 665-692 billion Euros per year between the budgets in scenarios with the endogenous method (see also upper plot in Figure S26). The endogenous method leads to lower total costs, especially with the +1.5°C budget, since cost reductions due to faster decarbonisation are better represented compared to the sequential and exogenous methods (see Figure S27, Figure S28 and Figure S29).

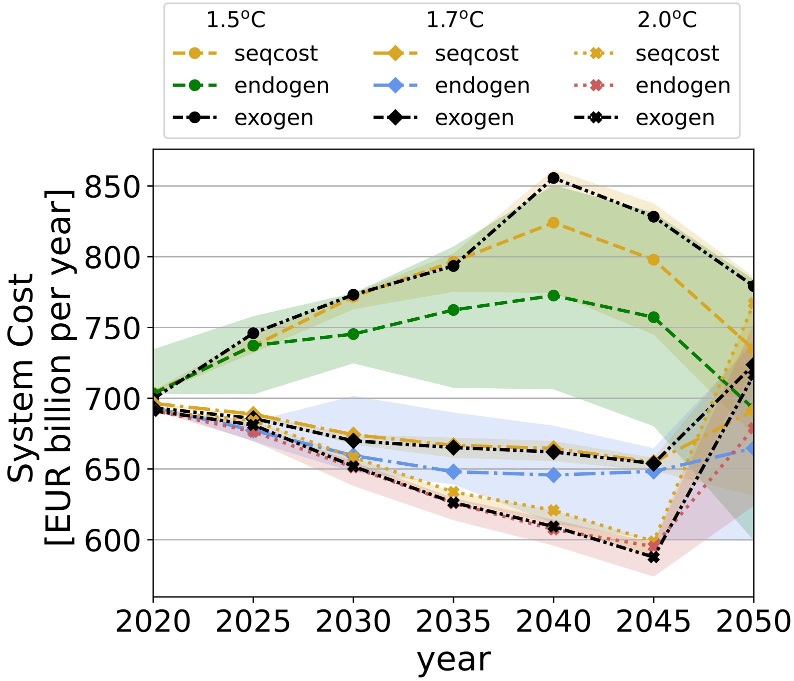

Fig. S25: Annualised total system costs without estimated cost of climate change damage. Contour area shows scenarios in which the learning rate is varied by ±10%. If endogenous learning is considered, total system costs in 2050 are of comparable size for the different budgets.

Annualised total system cost difference between the three budgets

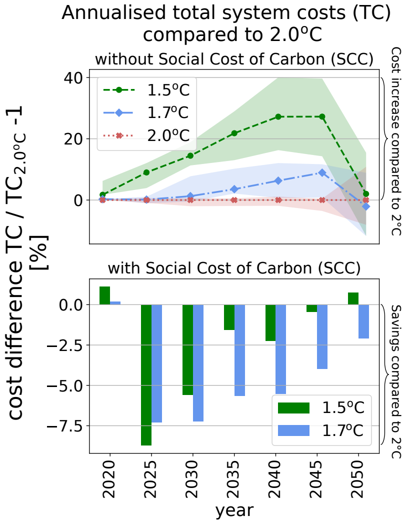

Fig. S26: Total annualised system costs compared to the endogenous +2°C budget scenario with and without estimated costs of climate change damage with social cost of carbon (SCC) of 120 € per tonne CO_2.

Annualised total system cost difference between the methods

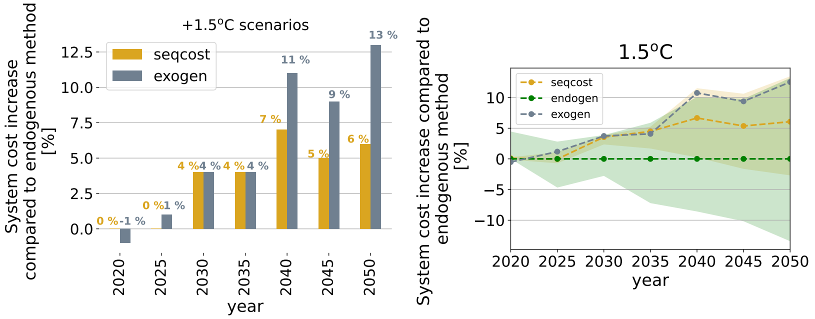

(a) base learning rate

(b) with varied learning rate

Fig. S27: Difference in total system costs for +1.5°C scenarios compared to the endogenous method with base learning rates. Exogenous (*exogen*) and sequential (*seqcost*) method result in higher total annualised system costs compared to the endogenous scenarios. The contour area shows scenarios which vary the base learning rate by ±10%.

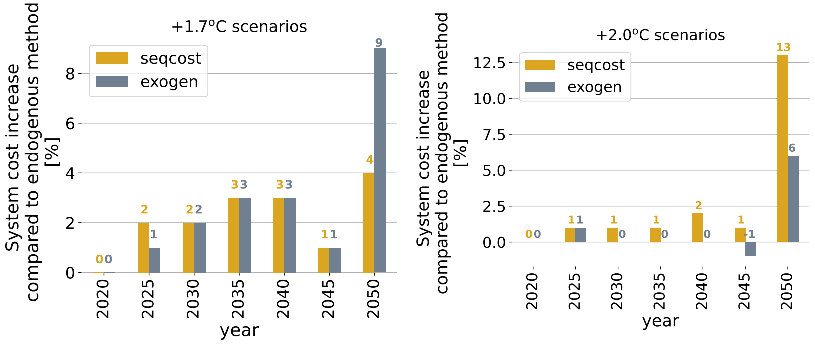

(a) +1.7°C

(b) +2.0°C

Fig. S28: Difference in total system costs for +1.7°C and +2.0°C scenarios compared to the endogenous method with base learning rates. Exogenous (*exogen*) and sequential (*seqcost*) method result in higher total annualised system costs compared to the endogenous scenarios.

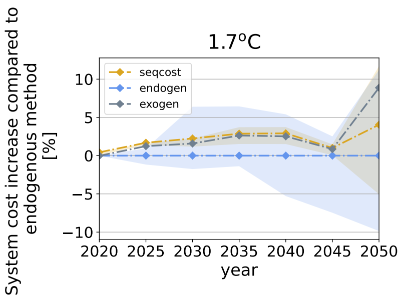

(a) +1.7°C

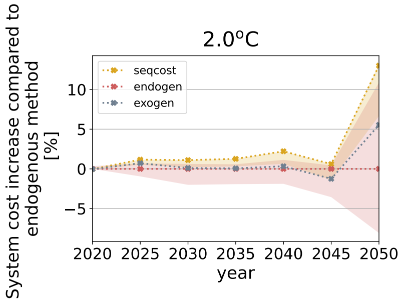

(b) +2.0°C

Fig. S29: Difference in total system costs for +1.7°C and +2.0°C scenarios compared to the endogenous method. Contour area shows scenarios which vary the base learning rate by ±10%. Exogenous (*exogen*) and sequential (*seqcost*) method result in higher total annualised system costs compared to the endogenous scenarios.

Energy balances

In the following, we show the energy balances for all considered energy carriers (e.g. hydrogen). We compare the three different budgets with the endogenous method and base learning rates, as well as the three different methods to model technology-learning (endogenous, sequential cost and exogenous) for the +1.7°C scenarios.

Hydrogen

The stricter the CO_2 budget, the more hydrogen is used, especially in the period between 2025-2045 (see Figure S30). In scenarios with a +1.5°C budget, most of the hydrogen is already produced as green hydrogen in 2025. With a +2.0°C budget, production switches from grey to green hydrogen later in 2040. The option of producing blue hydrogen is not used in any of the scenarios. In all scenarios, hydrogen is used in 2050 for the production of synthetic fuels and feedstocks, for methanation and in the transport sector. A small part is converted back into electricity in fuel cells. However, the production of synthetic fuels and the methanation of hydrogen starts earlier in scenarios with a +1.5°C budget, from 2030, with a +1.7°C budget from 2045 and with a +2.0°C budget from 2050.

With the endogenous method, the production changes from grey to green hydrogen at an earlier point in time compared to the sequential and exogenous method. Thus, with the endogenous method, the majority of hydrogen is already produced green in 2035, while with the sequential and exogenous method, the majority of hydrogen is still produced via SMR. The volume produced also differs between the methods. With the endogenous method, 1600 TWh_{H_2} of hydrogen are already produced in 2045, while with the sequential and exogenous method only 1000 TWh_{H_2} are produced. The larger volume of hydrogen in scenarios with the endogenous method is mainly used for the production of synthetic fuels.

Electricity

A larger amount of electricity is produced with a tighter budget (see Figure S31). For example, in 2040 about 10700 TWh of electricity are produced with the +1.5°C budget, 6600 TWh with the +1.7°C budget and 6100 TWh with the +2.0°C budget. The larger amount of electricity produced in scenarios with a +1.5°C budget compared to the +2.0°C budget is produced in renewable energy (for example +3000 TWh in 2030), while the production of conventional energy sources is lower (for example -1500 TWh in 2030). This additional electricity is mainly used for the production of green hydrogen via electrolysis and heat pumps which are employed earlier compared to the +2.0°C budget (see Figure S32).

In scenarios with the endogenous method up to 1400 TWh more electricity are produced compared to the exogenous and sequential method. More electricity is generated by PV with the endogenous learning compared to the other two methods, as the investment costs decrease to a greater extent. The electricity demand is higher with the endogeouns method since the endogenous method produces hydrogen via electrolysis at higher volumes and at an earlier point in time.

Carbon dioxide

At the beginning of the modelling period in 2020, CO_2 emissions are comparable between the three budgets (see Figure S33). Large parts of the emissions are generated in the transport sector (31%), in the combustion of fossil fuels (coal 21%, gas 15% and oil 11%), in the heating sector (oil and gas boilers 12%) and in industrial processes (7%). 1% of total CO_2 emissions are caused by the production of grey hydrogen. The stricter the available CO_2 budget, the faster annual emissions are reduced. In all scenarios, coal and oil are replaced by other energy sources. In the +1.5°C scenario coal and oil are substituted by renewable energies, in the +1.7°C and +2.0°C scenarios in a transition phase with gas (see Figure S34 and Figure S35). In 2030, coal-fired power plants are largely decommissioned in the +1.5°C and +1.7°C scenarios (share of total emissions is <1%), while in the +2.0°C scenario they continue to contribute about 6% to total CO_2 emissions. Emissions from land transport are reduced as internal combustion engines are continuously replaced by fuel and electric vehicles according to the exogenously defined transformation path (see Figure S36). Fossil oil is replaced by synthetic liquid hydrocarbons via the Fischer-Tropsch process. In the +1.5°C scenario this replacements start in 2035, in the +1.7°C scenario in 2045 and in the +2.0°C scenario in 2050. The produced synthetic liquid hydro carbons are mainly used as fuel for aircraft and naphtha production in industry (see Figure S37). In the +1.5°C scenario, it is also used for synthetic fuels in land transport. In 2050, CO_2 emissions are net-zero for all budgets. Remaining emissions from industrial processes and oil are offset by bioenergy with carbon capture and storage (BECCS, see Figure S38 and Figure S39), carbon capture directly from the industrial process (see Figure S40) and DAC. This leads to even slightly net-negative emissions in scenarios with a +1.5°C budget. The CO_2 sequestration potential of 200 Mt/CO_2 per year is completely exploited for all budgets in 2050 (see Figure S41).

The total annual CO_2 emissions for the +1.7°C budget are similar between the three methods. With the endogenous method, fossil oil for the production of naphtha and aviation fuels is produced to a larger extent already in 2045 via the Fischer Tropsch process compared to the other two methods (see Figure S37). This is due to the cheaper production of green hydrogen. In addition, with the endogenous method, some industrial processes are already converted to capture the generated CO_2 in 2040. Whereas with the sequential and exogenous method, carbon capture of process emissions is used from 2045 onwards and to a lesser extent (see Figure S40).

Heat

The heating sector is divided into rural areas (see Figure S42), urban areas with individual heating (see Figure S43) and urban areas with district heating (see Figure S44). In all scenarios, the heat sector is mostly electrified by 2050. In the district heating network, CHPs which run with biomass, biogas or synthetic gas, as well as hydrogen are also used. The stricter the CO_2 budget, the faster the shift from fossil fuels such as gas boilers, to heat pumps and resistive heaters. In 2030, in scenarios with a +1.5°C budget, more than half of the demand in rural areas is already met by heat pumps or

resistive heaters, while in the +1.7°C and +2.0°C budget most of the demand is met by gas boilers. In district heating networks, the share of CHPs is higher in +1.7°C and +2.0°C scenarios compared to the +1.5°C budget. With a +1.5°C budget, heat pumps are used to a larger extent which do not emit additional CO_2.

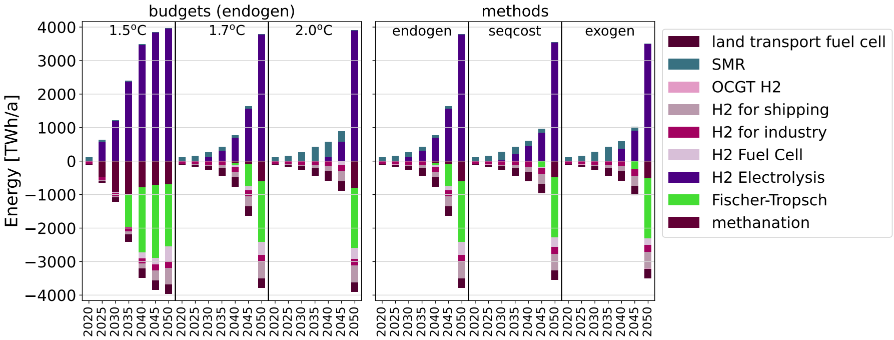

Fig. S30: Energy balance for hydrogen. Supply site is positive, usage is negative. Left plot shows the three different budgets with the endogenous method, right plot the three different methods endogenous (*endogen*), sequential cost (*seqcost*) and exogenous (*exogen*). Values are also displayed in Tables S3, S4.

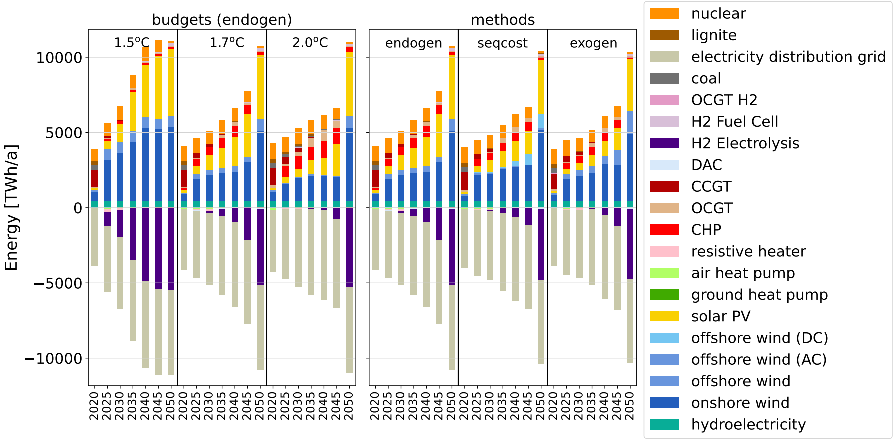

Fig. S31: Energy balance for electricity (transmission level). Supply site is positive, usage is negative. Left plot shows the three different budgets with the endogenous method, right plot the three different methods endogenous (*endogen*), sequential cost (*seqcost*) and exogenous (*exogen*). Values are also displayed in Tables S5, S6.

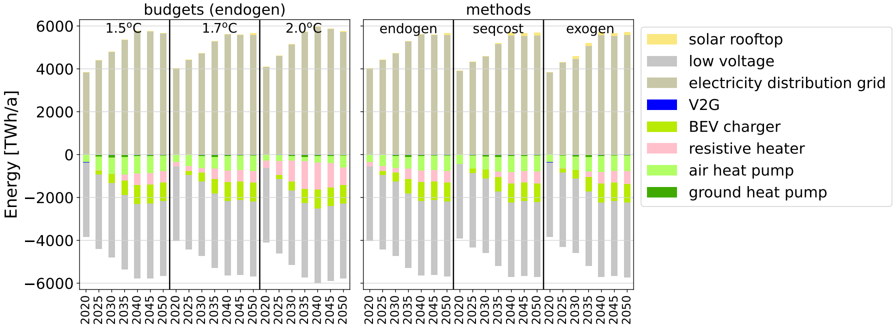

Fig. S32: Energy balance for electricity (distribution level). Supply site is positive, usage is negative. Left plot shows the three different budgets with the endogenous method, right plot the three different methods endogenous (*endogen*), sequential cost (*seqcost*) and exogenous (*exogen*). Values are also displayed in Tables S7, S8.

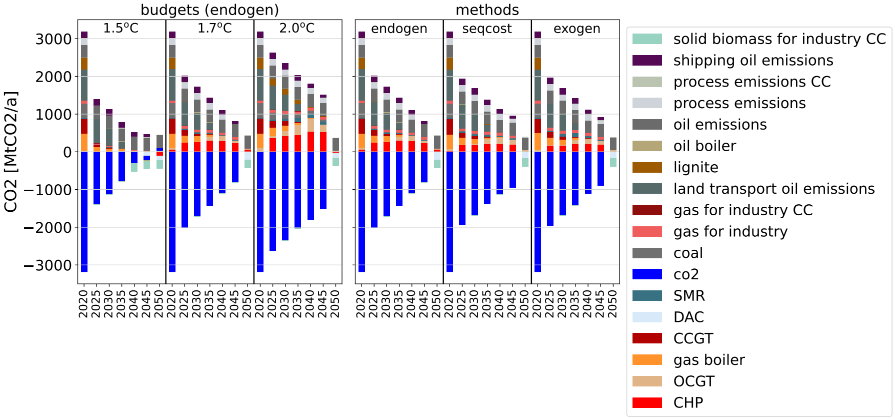

Fig. S33: Energy balance for carbon dioxide. Supply site is positive, usage is negative. Left plot shows the three different budgets with the endogenous method, right plot the three different methods endogenous (*endogen*), sequential cost (*seqcost*) and exogenous (*exogen*). Values are also displayed in Tables S9, S10.

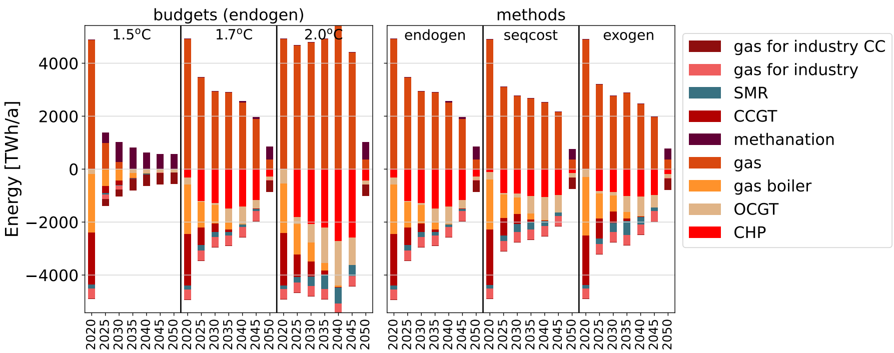

Fig. S34: Energy balance for methane. Supply site is positive, usage is negative. Left plot shows the three different budgets with the endogenous method, right plot the three different methods endogenous (*endogen*), sequential cost (*seqcost*) and exogenous (*exogen*). Values are also displayed in Tables S11, S12.

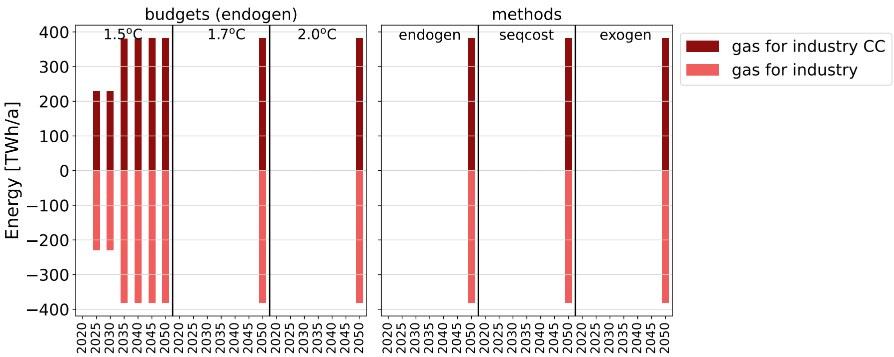

Fig. S35: Energy balance for methane for industry. Supply site is positive, usage is negative. Left plot shows the three different budgets with the endogenous method, right plot the three different methods endogenous (*endogen*), sequential cost (*seqcost*) and exogenous (*exogen*). Values are also displayed in Tables S13, S14.

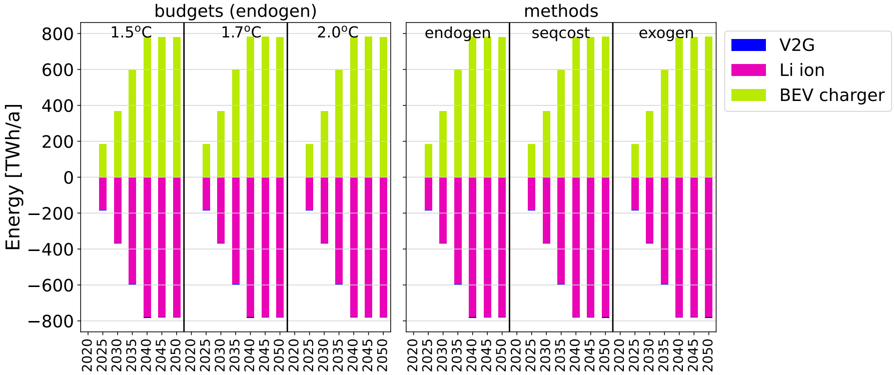

Fig. S36: Energy balance for electric vehicles. Supply site is positive, usage is negative. Left plot shows the three different budgets with the endogenous method, right plot the three different methods endogenous (*endogen*), sequential cost (*seqcost*) and exogenous (*exogen*). Values are also displayed in Tables S15, S16. The share of electric vehicles is exogenously fixed for all scenarios.

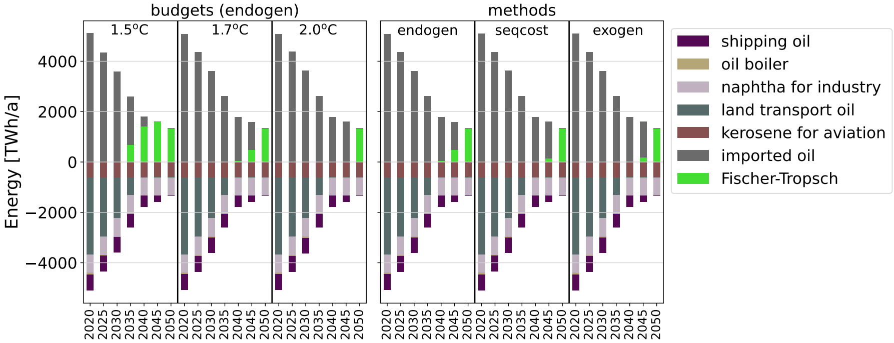

Fig. S37: Energy balance for oil. Supply site is positive, usage is negative. Left plot shows the three different budgets with the endogenous method, right plot the three different methods endogenous (*endogen*), sequential cost (*seqcost*) and exogenous (*exogen*). Values are also displayed in Tables S17, S18.

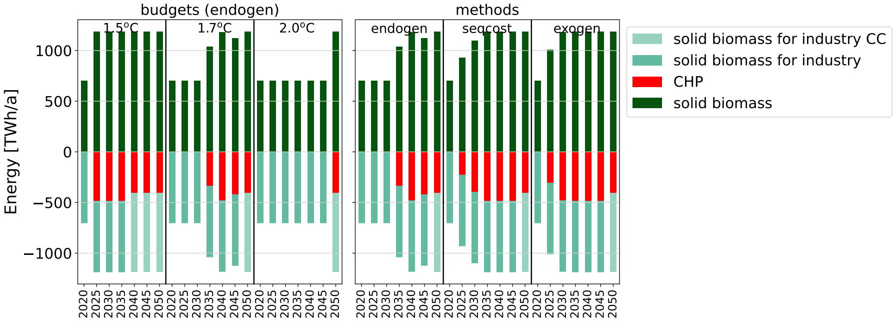

Fig. S38: Energy balance for solid biomass. Supply site is positive, usage is negative. Left plot shows the three different budgets with the endogenous method, right plot the three different methods endogenous (*endogen*), sequential cost (*seqcost*) and exogenous (*exogen*). Values are also displayed in Tables S19, S20.

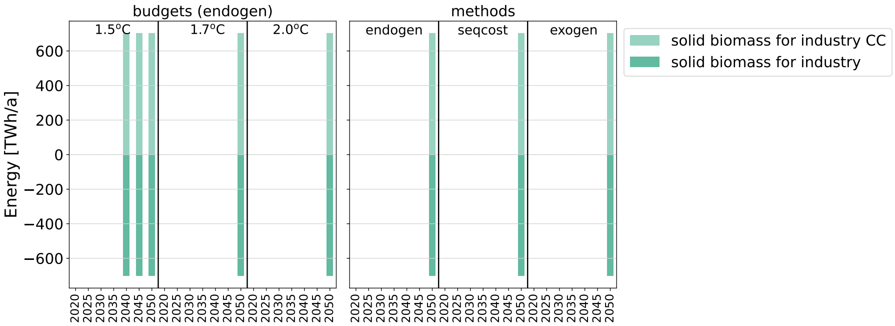

Fig. S39: Energy balance for solid biomass for industry. Supply site is positive, usage is negative. Left plot shows the three different budgets with the endogenous method, right plot the three different methods endogenous (*endogen*), sequential cost (*seqcost*) and exogenous (*exogen*). Values are also displayed in Tables S21, S22.

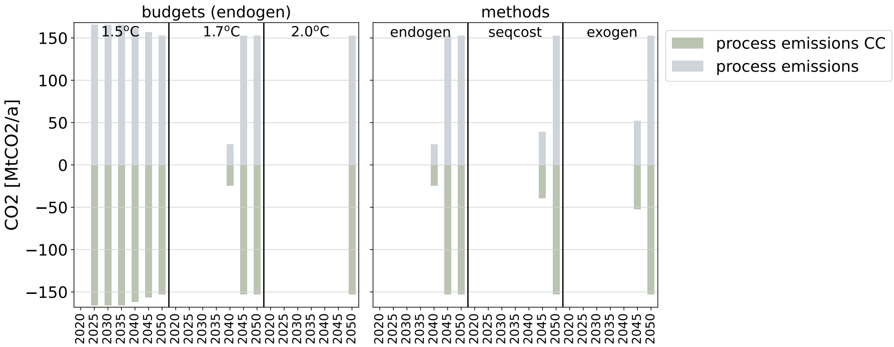

Fig. S40: Energy balance for process emissions. Supply site is positive, usage is negative. Left plot shows the three different budgets with the endogenous method, right plot the three different methods endogenous (*endogen*), sequential cost (*seqcost*) and exogenous (*exogen*). Values are also displayed in Tables S23, S24.

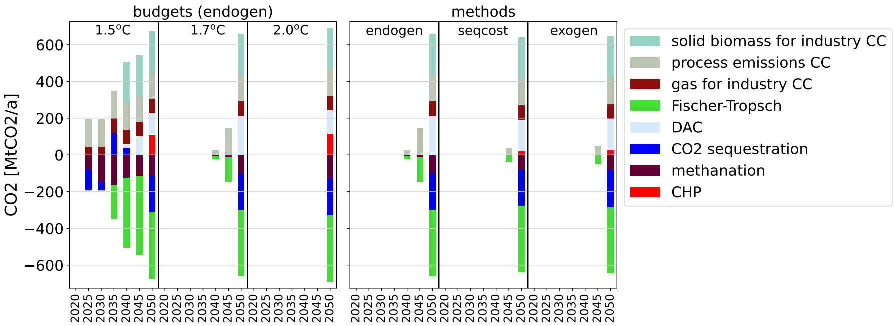

Fig. S41: Energy balance for carbon dioxide storage. Supply site is positive, usage is negative. Left plot shows the three different budgets with the endogenous method, right plot the three different methods endogenous (*endogen*), sequential cost (*seqcost*) and exogenous (*exogen*). Values are also displayed in Tables S25, S26.

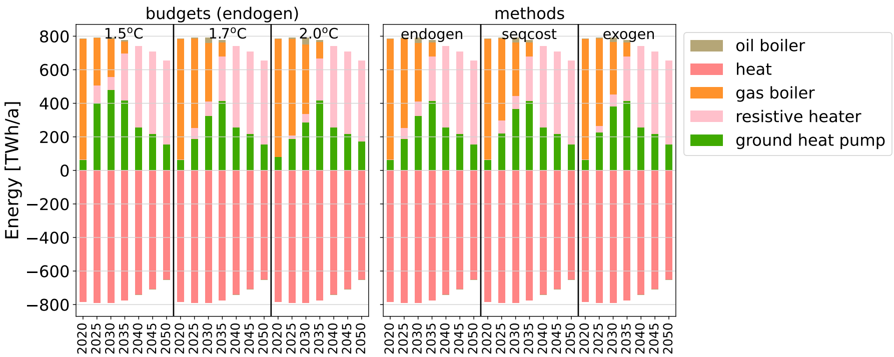

Fig. S42: Energy balance for rural heat. Supply site is positive, usage is negative. Left plot shows the three different budgets with the endogenous method, right plot the three different methods endogenous (*endogen*), sequential cost (*seqcost*) and exogenous (*exogen*). Values are also displayed in Tables S27, S28.

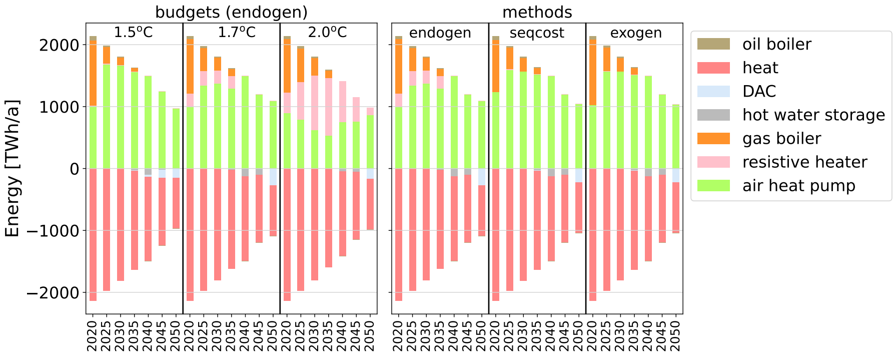

Fig. S43: Energy balance for urban individual heating. Supply site is positive, usage is negative. Left plot shows the three different budgets with the endogenous method, right plot the three different methods endogenous (*endogen*), sequential cost (*seqcost*) and exogenous (*exogen*). Values are also displayed in Tables S29, S30.

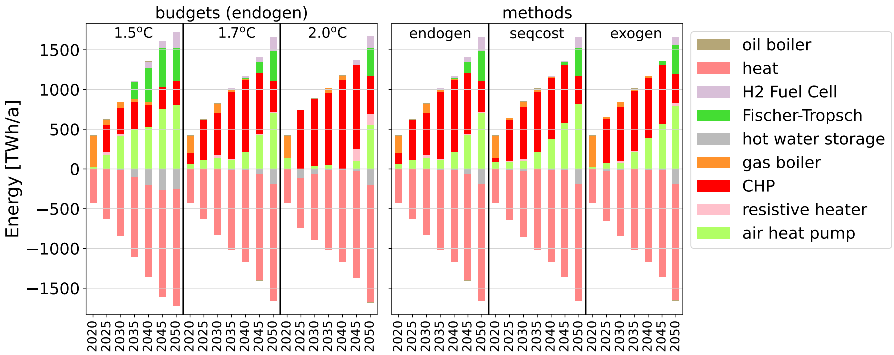

Fig. S44: Energy balance for urban district heating. Supply site is positive, usage is negative. Left plot shows the three different budgets with the endogenous method, right plot the three different methods endogenous (*endogen*), sequential cost (*seqcost*) and exogenous (*exogen*). Values are also displayed in Tables S31, S32.

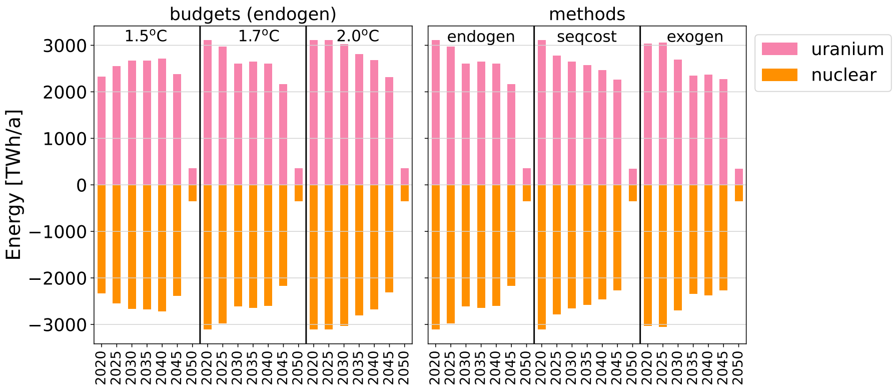

Fig. S45: Energy balance for uranium. Supply site is positive, usage is negative. Left plot shows the three different budgets with the endogenous method, right plot the three different methods endogenous (*endogen*), sequential cost (*seqcost*) and exogenous (*exogen*). Values are also displayed in Tables S33, S34.

Electrolysis duration curve

The full load hours of electrolysis vary between a maximum of 1032-1334 hours per year between the +2.0°C and +1.5°C budget. In the +1.5°C budget, the electrolysis is operated on average in more hours per year (77%) compared to the +2.0°C budget (16%). In later years, electrolysis tends to be operated in more hours in all budgets (see Figure S46).

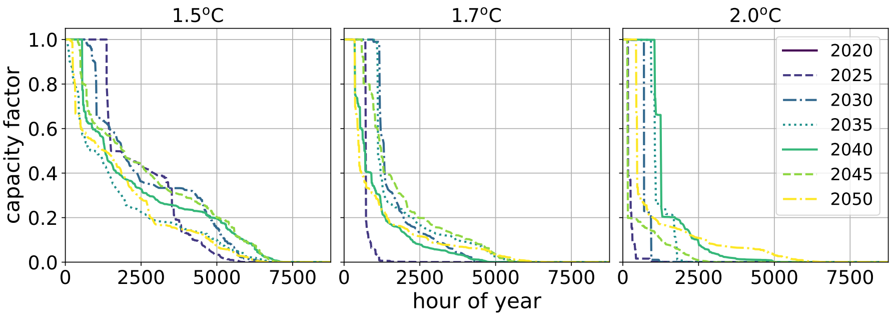

Fig. S46: Duration curve of hydrogen electrolysis.

Technology assumptions

The technology assumptions are listed in the tables below. Overnight costs $c_{\text{annualised}}$ are calculated for each assets based on investment costs *inv*, fixed operational and maintenance cost (FOM) FOM, lifetime n and discount rate τ.

$$f_{\text{annuity}} = (1-(1+\tau)^{-n})/\tau \tag{7}$$

$$c_{\text{annualised}} = (f_{\text{annuity}} + FOM/100) \cdot inv \tag{8}$$

The assumed discount rate in this study is 7%. If variable operational and maintenance cost (VOM) or lifetime are not listed for a technology, zero costs and 25 years are assumed accordingly. The efficiencies for all thermal plants are given in units of lower heating value. The investment cost include equipment and installation cost. Infrastructure and connection expenditures within a plant are included in the investment costs. All financial data is given in Euro at the 2015-level. All our technology assumptions with a detailed description of the sources can be found openly available in the Github repository `technology-data`[32], Version 0.3.0 is used for this study.

build year	2020	2025	2030	2035	2040	2045	2050
electrolysis	650	550	450	375	300	275	250
offshore wind	1748	1661	1573	1510	1447	1432	1416
onshore wind	1119	1077	1036	1007	978	970	963
solar PV	529	453	376	353	330	316	302

Table S1: Investment costs [€/kW] in the exogenous scenarios for the learning technologies, based on[21]. For wind and solar further grid connection costs are added.

technology	parameter	2020	2025	2030	2035	2040	2045	2050	unit	source
Air-sourced heat pump central	FOM	0.21	0.21	0.23	0.23	0.23	0.23	0.23	% of investment/year	[21]
	VOM	2.19	2.19	2.51	2.35	2.19	2.43	2.67	EUR/MWh_{th}	[21]
	efficiency[1]	340	350	360	362	365	368	370	%	[21]
	investment	951	951	856	856	856	856	856	EUR/kW_{th}	[21]
	lifetime	25	25	25	25	25	25	25	years	[21]
Air-sourced heat pump decentral	FOM	2.96	2.98	3	3.03	3.07	3.10	3.14	% of investment/year	[21]
	efficiency[1]	340	350	360	365	370	375	380	%	[21]
	investment	940	895	850	828	805	782	760	EUR/kW_{th}	[21]
	lifetime	18	18	18	18	18	18	18	years	[21]
Battery inverter	FOM	0.20	0.25	0.34	0.42	0.54	0.68	0.90	% of investment/year	[21]
	efficiency	95	96	96	96	96	96	96	%	[21]
	investment	270	215	160	130	100	80	60	EUR/kW	[21]
	lifetime	10	10	10	10	10	10	10	years	[21]
Battery storage	investment	232	187	142	118	94	84	75	EUR/kWh	[21]
	lifetime	20	22	25	28	30	30	30	years	[21]
Biogas upgrading	FOM	2.51	2.50	2.49	2.50	2.50	2.50	2.51	% of investment/year	[21]
	VOM	3.69	3.44	3.18	3.31	3.43	3.56	3.68	EUR/MWh input	[21]
	investment	423	402	381	372	362	352	343	EUR/kW input	[21]
	lifetime	15	15	15	15	15	15	15	years	[21]
CCGT	FOM	3.33	3.34	3.35	3.33	3.30	3.28	3.25	% of investment/year	[21]
	VOM	4.40	4.30	4.20	4.15	4.10	4.05	4	EUR/MWh	[21]
	efficiency	56	57	58	58	59	60	60	%	[21]
	investment	880	855	830	822	815	808	800	EUR/kW	[21]
	lifetime	25	25	25	25	25	25	25	years	[21]
CHP biomass	FOM	3.61	3.60	3.58	3.57	3.56	3.55	3.54	% of investment/year	[21]
	VOM	2.11	2.10	2.10	2.10	2.10	2.10	2.10	EUR/MWh_{el}	[21]
	efficiency	30	30	30	30	30	30	30	%	[21]
	investment	3381	3296	3210	3136	3061	2987	2912	EUR/kW_{el}	[21]
	lifetime	25	25	25	25	25	25	25	years	[21]
CHP gas	FOM	3.31	3.31	3.32	3.35	3.39	3.42	3.46	% of investment/year	[21]
	VOM	4.40	4.30	4.20	4.15	4.10	4.05	4	EUR/MWh	[21]
	efficiency	40	40	41	42	42	42	43	%	[21]
	investment	590	575	560	550	540	530	520	EUR/kW	[21]
	lifetime	25	25	25	25	25	25	25	years	[21]
CHP solid biomass	FOM	4.12	4.11	4.10	4.07	4.04	4.01	3.98	% of investment/year	[21]
	VOM	1.86	1.86	1.85	1.86	1.87	1.88	1.88	EUR/MWh_{el}	[21]
	efficiency	29	29	29	29	29	29	29	%	[21]
	investment	3007	2929	2851	2817	2783	2749	2714	EUR/kW_{el}	[21]
	lifetime	25	25	25	25	25	25	25	years	[21]
CHP solid biomass with Carbon Capture	FOM	3	3	3	3	3	3	3	% of investment/year	[21]
	investment	3300	3000	2700	2550	2400	2200	2000	$EUR/(kg_{CO_2}/h)$	[21]
	lifetime	25	25	25	25	25	25	25	years	[21]
CO_2 storage tank	FOM	1	1	1	1	1	1	1	% of investment/year	[33]
	investment	2528	2528	2528	2528	2528	2528	2528	EUR/t_{CO_2}	[33]
	lifetime	25	25	25	25	25	25	25	years	[33]
Cement capture	FOM	3	3	3	3	3	3	3	% of investment/year	[21]
	investment	3000	2800	2600	2400	2200	2000	1800	$EUR/(kg_{CO_2}/h)$	[21]
	lifetime	25	25	25	25	25	25	25	years	[21]
Coal power plant	FOM	1.60	1.60	1.60	1.60	1.60	1.60	1.60	% of investment/year	[34]
	VOM	3.50	3.50	3.50	3.50	3.50	3.50	3.50	EUR/MWh_{el}	[34]
	efficiency	33	33	33	33	33	33	33	%	[34]
	investment	3846	3846	3846	3846	3846	3846	3846	EUR/kW_{el}	[34]
	lifetime	40	40	40	40	40	40	40	years	[34]
DAC (direct-air capture)	FOM	4.95	4.95	4.95	4.95	4.95	4.95	4.95	% of investment/year	[21]
	investment	7000	7000	6000	5500	5000	4500	4000	$EUR/(kg_{CO_2}/h)$	[21]
	lifetime	20	20	20	20	20	20	20	years	[21]
Electricity distribution grid	FOM	2	2	2	2	2	2	2	% of investment/year	[21]
	investment	500	500	500	500	500	500	500	EUR/kW	[21]
	lifetime	40	40	40	40	40	40	40	years	[21]
Electricity grid connection [2]	FOM	2	2	2	2	2	2	2	% of investment/year	[21]
	investment	140	140	140	140	140	140	140	EUR/kW	[21]
	lifetime	40	40	40	40	40	40	40	years	[21]
Electrolysis [3]	FOM	2	2	2	2	2	2	2	% of investment/year	[21]
	efficiency	66	67	68	70	72	73	75	%	[21]
	investment	650	550	450	375	300	275	250	EUR/kW_{el}	[21]
	lifetime	25	28	30	31	32	34	35	years	[21]
Fischer-Tropsch	FOM	3	3	3	3	3	3	3	% of investment/year	[35]
	VOM	5.30	4.75	4.20	3.70	3.20	2.65	2.10	EUR/MWh_{FT}	[21]

[1] Efficiencies of heat pumps are time-dependent based on ambient temperature.
[2] Grid connection costs for solar and onshore wind. For offshore wind these are calculated separately depending on the connection type. Electricity grid connection costs do not underlay any learning.
[3] Technology assumptions for Alkaline Electrolysis 100 MW plant.

technology	parameter	2020	2025	2030	2035	2040	2045	2050	unit	source
Fischer-Tropsch	efficiency	65	68	70	72	73	74	75	%	21
	investment	2100	1850	1600	1350	1100	1000	900	EUR/kW$_{FT}$/year	21
	lifetime	25	25	25	25	25	25	25	years	21
Fuel cell	FOM	5	5	5	5	5	5	5	% of investment/year	21
	efficiency	50	50	50	50	50	50	50	%	21
	investment	1300	1200	1100	1025	950	875	800	EUR/kW$_{el}$	21
	lifetime	10	10	10	10	10	10	10	years	21
Gas boiler central	FOM	3.25	3.50	3.80	3.70	3.60	3.50	3.40	% of investment/year	21
	VOM	1.10	1.05	1	1	1	1	1	EUR/MWh$_{th}$	21
	efficiency	103	103	104	104	104	104	104	%	21
	investment	60	55	50	50	50	50	50	EUR/kW$_{th}$	21
	lifetime	25	25	25	25	25	25	25	years	21
Gas boiler connection	investment	195	190	186	181	177	172	168	EUR/kW$_{th}$	21
	lifetime	50	50	50	50	50	50	50	years	21
Gas boiler decentral	FOM	6.56	6.62	6.69	6.70	6.71	6.72	6.73	% of investment/year	21
	efficiency	97	98	98	98	98	99	99	%	21
	investment	312	304	297	290	283	276	269	EUR/kW$_{th}$	21
	lifetime	20	20	20	20	20	20	20	years	21
Geothermal	FOM	2.36	2.36	2.36	2.36	2.36	2.36	2.36	% of investment/year	36
	efficiency	24	24	24	24	24	24	24	%	36
	investment	3392	3392	3392	3392	3392	3392	3392	EUR/kW$_{el}$	36
	lifetime	40	40	40	40	40	40	40	years	37
Ground-sourced heat pump decentral	FOM	1.85	1.84	1.82	1.86	1.90	1.94	1.99	% of investment/year	21
	efficiency[1]	380	385	390	394	398	401	405	%	21
	investment	1500	1450	1400	1350	1300	1250	1200	EUR/kW$_{th}$	21
	lifetime	20	20	20	20	20	20	20	years	21
H_2 storage tank incl. compressor	FOM	1.05	1.08	1.11	1.39	1.85	1.87	1.90	% of investment/year	21
	investment	57	51	45	36	27	24	21	EUR/kWh	21
	lifetime	25	28	30	30	30	30	30	years	21
H_2 storage underground	FOM	0	0	0	0	0	0	0	% of investment/year	21
	VOM	0	0	0	0	0	0	0	EUR/MWh	21
	investment	3	2	2	2	2	1	1	EUR/kWh	21
	lifetime	100	100	100	100	100	100	100	years	21
Home battery inverter	FOM	0.20	0.25	0.34	0.42	0.54	0.68	0.90	% of investment/year	21
	efficiency	95	96	96	96	96	96	96	%	21
	investment	377	304	228	187	145	116	87	EUR/kW	21
	lifetime	10	10	10	10	10	10	10	years	21
Home battery storage	investment	324	265	203	170	136	123	109	EUR/kWh	21
	lifetime	20	22	25	28	30	30	30	years	21
Industrial heat pump medium temperature	FOM	0.11	0.11	0.11	0.11	0.11	0.11	0.10	% of investment/year	21
	VOM	3.26	3.23	3.20	3.21	3.22	3.17	3.12	EUR/MWh	21
	efficiency	255	262	270	275	280	282	285	%	21
	investment	871	825	779	754	730	715	700	EUR/kW	21
	lifetime	20	20	20	20	20	20	20	years	21
Lignite power plant	FOM	1.60	1.60	1.60	1.60	1.60	1.60	1.60	% of investment/year	34
	VOM	3.50	3.50	3.50	3.50	3.50	3.50	3.50	EUR/MWh$_{el}$	34
	efficiency	33	33	33	33	33	33	33	%	34
	investment	3846	3846	3846	3846	3846	3846	3846	EUR/kW$_{el}$	34
	lifetime	40	40	40	40	40	40	40	years	34
Methanation	FOM	4	4	4	4	4	4	4	% of investment/year	35
	efficiency	80	80	80	80	80	80	80	%	38
	investment	278	278	278	252	226	226	226	EUR/kW$_{CH_4}$	35
	lifetime	30	30	30	30	30	30	30	years	35
Nuclear power plant	FOM	1.40	1.40	1.40	1.40	1.40	1.40	1.40	% of investment/year	34
	VOM	3.50	3.50	3.50	3.50	3.50	3.50	3.50	EUR/MWh$_{el}$	34
	efficiency	33	33	33	33	33	33	33	%	34
	investment	7940	7940	7940	7940	7940	7940	7940	EUR/kW$_{el}$	34
	lifetime	40	40	40	40	40	40	40	years	34
OCGT	FOM	1.78	1.78	1.78	1.78	1.79	1.80	1.80	% of investment/year	21
	VOM	4.50	4.50	4.50	4.50	4.50	4.50	4.50	EUR/MWh	21
	efficiency	40	40	41	42	42	42	43	%	21
	investment	454	445	435	429	424	418	412	EUR/kW	21
	lifetime	25	25	25	25	25	25	25	years	21

[1] Efficiencies of heat pumps are time-dependent based on ground temperature.

technology	parameter	2020	2025	2030	2035	2040	2045	2050	unit	source
Offshore Wind	FOM	2.29	2.29	2.29	2.29	2.29	2.29	2.29	% of investment/year	21
	VOM	2.97	2.82	2.67	2.56	2.46	2.43	2.40	EUR/MWh	21
	investment	1748	1661	1573	1510	1447	1432	1416	EUR/kW	21
	lifetime	27	28	30	30	30	30	30	years	21
Offshore wind-ac-connection-submarine	investment	2685	2685	2685	2685	2685	2685	2685	EUR/MW/km	21
Offshore wind-ac-connection-underground	investment	1342	1342	1342	1342	1342	1342	1342	EUR/MW/km	21
Offshore wind-ac-station	investment	250	250	250	250	250	250	250	EUR/kW$_{el}$	21
Offshore wind-dc-connection-submarine	investment	2000	2000	2000	2000	2000	2000	2000	EUR/MW/km	39
Offshore wind-dc-connection-underground	investment	1000	1000	1000	1000	1000	1000	1000	EUR/MW/km	40
Offshore wind-dc-station	investment	400	400	400	400	400	400	400	EUR/kW$_{el}$	40
Oil boiler decentral	FOM	2	2	2	2	2	2	2	% of investment/year	38
	efficiency	90	90	90	90	90	90	90	%	38
	investment	156	156	156	156	156	156	156	EUR/kWth	38
	lifetime	20	20	20	20	20	20	20	years	38
Onshore Wind	FOM	1.25	1.23	1.22	1.20	1.19	1.18	1.18	% of investment/year	21
	VOM	1.50	1.42	1.35	1.30	1.24	1.23	1.22	EUR/MWh	21
	investment	1119	1077	1036	1007	978	970	963	EUR/kW	21
	lifetime	27	28	30	30	30	30	30	years	21
PHS	FOM	1	1	1	1	1	1	1	% of investment/year	36
	efficiency	75	75	75	75	75	75	75	%	36
	investment	2208	2208	2208	2208	2208	2208	2208	EUR/kW$_{el}$	36
	lifetime	80	80	80	80	80	80	80	years	37
Reservoir hydro	FOM	1	1	1	1	1	1	1	% of investment/year	36
	efficiency	90	90	90	90	90	90	90	%	36
	investment	2208	2208	2208	2208	2208	2208	2208	EUR/kW$_{el}$	36
	lifetime	80	80	80	80	80	80	80	years	37
Resistive heater central	FOM	1.53	1.61	1.70	1.66	1.62	1.58	1.53	% of investment/year	21
	VOM	0.90	0.95	1	1	1	1	1	EUR/MWh$_{th}$	21
	efficiency	99	99	99	99	99	99	99	%	21
	investment	70	65	60	60	60	60	60	EUR/kW$_{th}$	21
	lifetime	20	20	20	20	20	20	20	years	21
Resistive heater decentral	FOM	2	2	2	2	2	2	2	% of investment/year	41
	efficiency	90	90	90	90	90	90	90	%	41
	investment	100	100	100	100	100	100	100	EUR/kWhth	41
	lifetime	20	20	20	20	20	20	20	years	41
Run of river	FOM	2	2	2	2	2	2	2	% of investment/year	36
	efficiency	90	90	90	90	90	90	90	%	36
	investment	3312	3312	3312	3312	3312	3312	3312	EUR/kW$_{el}$	36
	lifetime	80	80	80	80	80	80	80	years	37
Solar PV (rooftop)	FOM	1.19	1.21	1.24	1.30	1.37	1.45	1.55	% of investment/year	21
	investment	1127	956	784	723	662	600	539	EUR/kW	21
	lifetime	30	30	30	30	30	30	30	years	21
Solar PV (utility-scale)	FOM	1.65	1.77	1.93	1.97	2.01	2.04	2.07	% of investment/year	21
	investment	529	453	376	353	330	316	302	EUR/kW	21
	lifetime	35	38	40	40	40	40	40	years	21
Solar thermal central	FOM	1.30	1.30	1.30	1.30	1.30	1.30	1.30	% of investment/year	42
	investment	270	270	270	270	270	270	270	EUR/Million m^2	42
	lifetime	20	20	20	20	20	20	20	years	42
Solar thermal decentral	FOM	1.40	1.40	1.40	1.40	1.40	1.40	1.40	% of investment/year	42
	investment	140	140	140	140	140	140	140	EUR/Million m^2	42
	lifetime	20	20	20	20	20	20	20	years	42
Steam Methane Reforming (SMR)	FOM	5	5	5	5	5	5	5	% of investment/year	21
	efficiency	76	76	76	76	76	76	76	%	37
	investment	493	493	493	493	493	493	493	EUR/kW$_{CH_4}$	21
	lifetime	30	30	30	30	30	30	30	years	37
SMR with Carbon Capture (CC)	FOM	5	5	5	5	5	5	5	% of investment/year	21
	efficiency	69	69	69	69	69	69	69	%	37
	investment	572	572	572	572	572	572	572	EUR/kW$_{CH_4}$	21
	lifetime	30	30	30	30	30	30	30	years	37
Water tank charger/discharger	efficiency	84	84	84	84	84	84	84	%	21
Water tank storage central	FOM	0.52	0.53	0.55	0.57	0.59	0.62	0.64	% of investment/year	21
	investment	1	1	1	1	1	0	0	EUR/kWhCapacity	21
	lifetime	20	22	25	25	25	25	25	years	21
Water tank storage decentral	FOM	1	1	1	1	1	1	1	% of investment/year	42
	investment	18	18	18	18	18	18	18	EUR/kWh	43
	lifetime	20	20	20	20	20	20	20	years	42

Table S2: Technology assumptions. Fixed operational and maintenance cost (FOM) are given as annual percentage of the investment costs. If variable operational and maintenance cost (VOM) are not listed for a technology, zero costs are assumed.

Energy balances tables

budget	+1.5°C							+1.7°C							+2.0°C						
year	2020	2025	2030	2035	2040	2045	2050	2020	2025	2030	2035	2040	2045	2050	2020	2025	2030	2035	2040	2045	2050
methanation	0	-478	-942	-1012	-786	-708	-701	0	0	0	0	-58	-84	-611	0	0	0	0	0	0	-807
Fischer-Tropsch	0	0	0	-961	-1946	-2192	-1844	0	0	0	0	-70	-661	-1799	0	0	0	0	0	0	-1788
H2 Electrolysis	0	590	1188	2385	3466	3833	3962	0	7	127	316	701	1565	3786	0	0	3	13	127	580	3900
H2 Fuel Cell	0	0	0	0	-172	-185	-444	0	0	0	0	-56	-131	-402	0	0	0	0	0	-129	-332
H2 for industry	-107	-107	-114	-134	-154	-180	-196	-107	-107	-114	-134	-154	-180	-196	-107	-107	-114	-134	-154	-180	-196
H2 for shipping	0	0	-25	-75	-151	-302	-503	0	0	-25	-75	-151	-302	-503	0	0	-25	-75	-151	-302	-503
SMR	107	50	22	16	19	9	2	107	155	141	113	63	67	0	107	162	265	416	454	306	0
land transport fuel cell	0	-55	-128	-220	-275	-275	-275	0	-55	-128	-220	-275	-275	-275	0	-55	-128	-220	-275	-275	-275

Table S3: Energy balance [TWh/a] for hydrogen for the three different budgets and the endogenous method. See also Figure S30.

method	endogen							exogen							seqcost						
year	2020	2025	2030	2035	2040	2045	2050	2020	2025	2030	2035	2040	2045	2050	2020	2025	2030	2035	2040	2045	2050
methanation	0	0	0	0	-58	-84	-611	0	0	0	0	0	0	-487	0	0	0	0	0	-11	-517
Fischer-Tropsch	0	0	0	0	-70	-661	-1799	0	0	0	0	0	-187	-1790	0	0	0	0	0	-240	-1791
H2 Electrolysis	0	7	127	316	701	1565	3786	0	0	38	211	447	854	3545	0	1	8	25	375	906	3497
H2 Fuel Cell	0	0	0	0	-56	-131	-402	0	0	0	0	-16	-17	-294	0	0	0	0	-7	-6	-204
H2 for industry	-107	-107	-114	-134	-154	-180	-196	-107	-107	-114	-134	-154	-180	-196	-107	-107	-114	-134	-154	-180	-196
H2 for shipping	0	0	-25	-75	-151	-302	-503	0	0	-25	-75	-151	-302	-503	0	0	-25	-75	-151	-302	-503
OCGT H2	0	0	0	0	0	0	0	0	0	0	0	0	0	0	0	0	0	0	0	0	-11
SMR	107	155	141	113	63	67	0	107	162	230	218	150	106	0	107	161	261	405	213	108	0
land transport fuel cell	0	-55	-128	-220	-275	-275	-275	0	-55	-128	-220	-275	-275	-275	0	-55	-128	-220	-275	-275	-275

Table S4: Energy balance [TWh/a] for hydrogen for the +1.7°C scenario and the three methods endogenous (*endogen*), exogenous (*exogen*) and sequential cost (*seqcost*). See also Figure S30.

	+1.5°C							+1.7°C							+2.0°C						
year	2020	2025	2030	2035	2040	2045	2050	2020	2025	2030	2035	2040	2045	2050	2020	2025	2030	2035	2040	2045	2050
hydroelectricity	466	460	458	459	457	457	455	465	463	452	455	453	453	456	467	464	460	455	454	451	457
onshore wind	561	2737	3181	3928	4820	4759	4943	436	1470	1709	1848	1961	2559	4637	611	1117	1528	1686	1677	1579	4853
offshore wind	142	737	744	734	747	723	725	106	340	382	376	372	331	800	70	85	85	76	74	116	787
solar PV	167	532	1222	2611	3483	4155	4469	166	514	982	1264	1918	2887	4249	161	407	512	961	1140	2129	4281
ground heat pump	-19	-9	-5	-2	0	0	0	-19	-16	-7	-2	0	0	0	-24	-16	-6	-2	0	0	0
air heat pump	-29	-123	-61	-19	0	0	0	-57	-88	-78	-27	0	0	0	-117	-49	-42	-29	0	0	0
resistive heater	-7	-175	-108	-44	0	0	0	-17	-98	-132	-39	0	0	0	-18	-45	-49	-26	0	0	0
CHP	0	140	140	140	118	117	106	126	482	516	698	711	596	238	0	729	832	882	1102	1045	287
OCGT	74	5	7	68	63	55	57	109	16	27	229	281	140	58	216	100	275	558	701	441	64
CCGT	1104	147	86	15	2	0	0	1090	380	185	51	7	0	0	1105	476	322	84	12	0	0
DAC	0	0	0	0	-10	-45	-52	0	0	0	0	0	0	-91	0	0	0	0	0	0	-55
H2 Electrolysis	0	-882	-1755	-3428	-4911	-5364	-5404	0	-11	-186	-455	-980	-2163	-5078	0	0	-4	-19	-176	-794	-5216
H2 Fuel Cell	0	0	0	0	86	93	222	0	0	0	0	28	65	201	0	0	0	0	0	64	166
coal	370	0	3	2	2	1	1	370	0	5	6	2	1	0	370	178	125	92	79	46	0
electricity distribution grid	-3836	-4411	-4792	-5347	-5752	-5736	-5639	-4037	-4434	-4720	-5279	-5612	-5584	-5587	-4104	-4617	-5148	-5736	-5964	-5855	-5741
lignite	239	0	2	1	0	0	0	239	0	4	5	0	0	0	239	148	112	90	17	16	0
nuclear	768	841	879	882	895	785	116	1025	982	861	872	858	715	116	1025	1025	999	926	883	763	116

Table S5: Energy balance [TWh/a] for the electricity transmission grid for the three different budgets and the endogenous method. See also Figure S31.

method	endogen							exogen							seqcost						
year	2020	2025	2030	2035	2040	2045	2050	2020	2025	2030	2035	2040	2045	2050	2020	2025	2030	2035	2040	2045	2050
hydroelectricity	465	463	452	455	453	453	456	466	464	452	453	455	451	454	466	465	460	457	455	457	458
onshore wind	436	1470	1709	1848	1961	2559	4637	358	1747	1767	2102	2240	2395	4730	348	1445	1651	1886	2440	2418	4455
offshore wind	106	340	382	376	372	331	800	70	70	57	55	48	24	0	164	305	389	471	540	938	1502
offshore wind (AC)	0	0	0	0	0	0	0	0	0	0	0	0	0	134	0	0	0	0	0	0	0
offshore wind (DC)	0	0	0	0	0	0	0	0	94	94	125	391	688	889	0	0	0	0	0	0	0
solar PV	166	514	982	1264	1918	2887	4249	141	409	810	1045	1298	1582	3650	141	358	453	701	991	1474	3437
ground heat pump	-19	-16	-7	-2	0	0	0	-19	-13	-6	-2	0	0	0	-19	-13	-6	-2	0	0	0
air heat pump	-57	-88	-78	-27	0	0	0	-62	-76	-64	-22	0	0	0	-33	-65	-51	-20	0	0	0
resistive heater	-17	-98	-132	-39	0	0	0	-7	-111	-113	-35	0	0	0	-7	-64	-104	-39	0	0	0
CHP	126	482	516	698	711	596	238	46	429	486	548	565	535	175	4	426	484	553	561	534	198
OCGT	109	16	27	229	281	140	58	116	24	60	280	366	280	79	118	31	58	255	311	202	65
CCGT	1090	380	185	51	7	0	0	1173	371	215	50	8	0	0	1047	421	240	57	8	0	0
DAC	0	0	0	0	0	0	-91	0	0	0	0	0	0	-74	0	0	0	0	0	0	-73
H2 Electrolysis	0	-11	-186	-455	-980	-2163	-5078	0	0	-56	-304	-627	-1181	-4743	0	-2	-11	-36	-521	-1244	-4674
H2 Fuel Cell	0	0	0	0	28	65	201	0	0	0	0	8	8	147	0	0	0	0	3	3	102
OCGT H2	0	0	0	0	0	0	0	0	0	0	0	0	0	0	0	0	0	0	0	0	4
coal	370	0	5	6	2	1	0	370	0	6	3	2	2	0	370	0	8	3	2	1	0
electricity distribution grid	-4037	-4434	-4720	-5279	-5612	-5584	-5587	-3915	-4326	-4585	-5149	-5569	-5530	-5557	-3837	-4316	-4464	-5062	-5573	-5532	-5588
lignite	239	0	4	5	0	0	0	239	0	3	2	0	0	0	239	0	3	2	0	0	0
nuclear	1025	982	861	872	858	715	116	1025	917	875	849	813	746	116	1000	1008	889	774	782	748	114

Table S6: Energy balance [TWh/a] for the electricity transmission grid for the +1.7°C scenario and the three methods endogenous (*endogen*), exogenous (*exogen*) and sequential cost (*seqcost*). See also Figure S31.

budget	+1.5°C							+1.7°C							+2.0°C						
year	2020	2025	2030	2035	2040	2045	2050	2020	2025	2030	2035	2040	2045	2050	2020	2025	2030	2035	2040	2045	2050
ground heat pump	0	-101	-126	-113	-71	-60	-42	0	-36	-82	-112	-71	-60	-42	0	-36	-72	-113	-71	-60	-48
air heat pump	-368	-639	-791	-826	-824	-810	-719	-345	-499	-540	-539	-683	-658	-722	-280	-262	-220	-205	-305	-343	-568
resistive heater	0	0	0	-275	-539	-544	-555	-223	-228	-223	-494	-539	-544	-555	-356	-649	-981	-1284	-1269	-1131	-805
BEV charger	0	-204	-408	-663	-870	-868	-868	0	-204	-410	-666	-870	-869	-869	0	-204	-408	-663	-866	-869	-868
V2G	0	0	0	0	3	1	1	0	0	2	3	3	2	2	0	0	0	0	0	2	1
electricity distribution grid	3836	4411	4792	5347	5752	5736	5639	4037	4434	4720	5279	5612	5584	5587	4104	4617	5148	5736	5964	5855	5741
low voltage	-3468	-3467	-3468	-3471	-3474	-3477	-3477	-3468	-3467	-3468	-3471	-3474	-3477	-3477	-3468	-3467	-3468	-3471	-3474	-3477	-3477
solar rooftop	0	0	0	0	22	22	22	0	0	0	0	22	22	77	0	0	0	0	22	22	24

Table S7: Energy balance [TWh/a] for electricity distribution level for the three different budgets and the endogenous method. See also Figure S32.

method	endogen							exogen							seqcost						
year	2020	2025	2030	2035	2040	2045	2050	2020	2025	2030	2035	2040	2045	2050	2020	2025	2030	2035	2040	2045	2050
ground heat pump	0	-36	-82	-112	-71	-60	-42	0	-47	-93	-112	-71	-60	-43	0	-49	-98	-112	-71	-60	-43
air heat pump	-345	-499	-540	-539	-683	-658	-722	-447	-607	-617	-679	-749	-713	-750	-369	-596	-622	-678	-753	-709	-736
resistive heater	-223	-228	-223	-494	-539	-544	-555	0	0	0	-270	-539	-544	-555	0	0	0	-270	-539	-551	-596
BEV charger	0	-204	-410	-666	-870	-869	-869	0	-204	-409	-663	-867	-866	-870	0	-204	-408	-663	-866	-866	-870
V2G	0	0	2	3	3	2	2	0	0	1	0	1	0	3	0	0	0	0	0	0	3
electricity distribution grid	4037	4434	4720	5279	5612	5584	5587	3915	4326	4585	5149	5569	5530	5557	3837	4316	4464	5062	5573	5532	5588
low voltage	-3468	-3467	-3468	-3471	-3474	-3477	-3477	-3468	-3467	-3468	-3471	-3474	-3477	-3477	-3468	-3467	-3468	-3471	-3474	-3477	-3477
solar rooftop	0	0	0	0	22	22	77	0	0	2	46	131	131	135	0	0	131	131	131	131	131

Table S8: Energy balance [TWh/a] for electricity distribution grid for the +1.7°C scenario and the three methods endogenous (*endogen*), exogenous (*exogen*) and sequential cost (*seqcost*). See also Figure S32.

budget	+1.5°C							+1.7°C							+2.0°C						
year	2020	2025	2030	2035	2040	2045	2050	2020	2025	2030	2035	2040	2045	2050	2020	2025	2030	2035	2040	2045	2050
CHP	0	0	0	0	0	-1	-109	63	241	258	297	283	234	58	0	365	416	441	544	517	-29
OCGT	37	3	3	32	30	26	27	54	8	13	109	134	67	28	108	50	137	268	337	210	31
gas boiler	442	127	85	35	5	0	0	375	191	138	50	1	0	0	377	231	145	59	11	0	0
CCGT	394	52	31	5	1	0	0	389	136	66	18	3	0	0	395	170	115	30	4	0	0
DAC	0	0	0	0	-22	-102	-118	0	0	0	0	0	0	-212	0	0	0	0	0	0	-127
SMR	28	13	6	4	5	2	0	28	41	37	30	17	18	0	28	43	70	110	119	80	0
co2	-3184	-1402	-1131	-782	-280	-133	72	-3184	-2018	-1720	-1436	-1111	-812	0	-3184	-2630	-2350	-2032	-1812	-1512	0
coal	381	0	3	2	2	1	1	381	0	5	6	2	1	0	381	183	129	95	82	48	0
gas for industry	76	30	30	0	0	0	0	76	76	76	76	76	76	0	76	76	76	76	76	76	0
gas for industry CC	0	5	5	8	8	8	5	0	0	0	0	0	0	4	0	0	0	0	0	0	4
land transport oil emissions	827	636	438	190	0	0	0	827	636	438	190	0	0	0	827	636	438	190	0	0	0
lignite	297	0	3	2	0	0	0	297	0	5	6	0	0	0	297	184	139	111	21	20	0
oil boiler	21	5	8	6	2	0	0	13	10	15	8	0	0	0	15	13	17	6	1	0	0
oil emissions	336	336	336	336	336	336	336	336	336	336	336	336	336	336	336	336	336	336	336	336	336
process emissions	174	7	4	0	0	0	0	174	172	170	166	137	4	0	174	172	170	166	162	157	0
process emissions CC	0	17	17	17	16	16	8	0	0	0	0	1	8	8	0	0	0	0	0	0	8
shipping oil emissions	170	170	161	144	119	68	0	170	170	161	144	119	68	0	170	170	161	144	119	68	0
solid biomass for industry CC	0	0	0	0	-222	-222	-222	0	0	0	0	0	0	-222	0	0	0	0	0	0	-222

Table S9: Energy balance [Mt CO_2/a] for carbon dioxide for the three different budgets and the endogenous method. See also Figure S33.

method	endogen							exogen							seqcost						
year	2020	2025	2030	2035	2040	2045	2050	2020	2025	2030	2035	2040	2045	2050	2020	2025	2030	2035	2040	2045	2050
CHP	63	241	258	297	283	234	58	23	182	186	202	211	195	8	2	169	172	204	208	195	13
OCGT	54	8	13	109	134	67	28	58	12	30	134	175	133	38	59	16	29	122	148	96	31
gas boiler	375	191	138	50	1	0	0	376	176	124	46	3	0	0	441	189	121	46	3	0	0
CCGT	389	136	66	18	3	0	0	419	132	77	18	3	0	0	374	150	86	20	3	0	0
DAC	0	0	0	0	0	0	-212	0	0	0	0	0	0	-171	0	0	0	0	0	0	-170
SMR	28	41	37	30	17	18	0	28	43	61	57	39	28	0	28	42	69	107	56	29	0
co2	-3184	-2018	-1720	-1436	-1111	-812	0	-3184	-1944	-1683	-1384	-1127	-959	0	-3184	-1966	-1684	-1424	-1114	-909	0
coal	381	0	5	6	2	1	0	381	0	6	3	3	2	0	381	0	8	3	2	1	0
gas for industry	76	76	76	76	76	76	0	76	76	76	76	76	76	0	76	76	76	76	76	76	0
gas for industry CC	0	0	0	0	0	0	4	0	0	0	0	0	0	4	0	0	0	0	0	0	4
land transport oil emissions	827	636	438	190	0	0	0	827	636	438	190	0	0	0	827	636	438	190	0	0	0
lignite	297	0	5	6	0	0	0	297	0	4	3	0	1	0	297	0	3	2	0	0	0
oil boiler	13	10	15	8	0	0	0	19	8	15	7	0	0	0	18	9	14	7	0	0	0
oil emissions	336	336	336	336	336	336	336	336	336	336	336	336	336	336	336	336	336	336	336	336	336
process emissions	174	172	170	166	137	4	0	174	172	170	166	162	117	0	174	172	170	166	162	104	0
process emissions CC	0	0	0	0	1	8	8	0	0	0	0	0	2	8	0	0	0	0	0	3	8
shipping oil emissions	170	170	161	144	119	68	0	170	170	161	144	119	68	0	170	170	161	144	119	68	0
solid biomass for industry CC	0	0	0	0	0	0	-222	0	0	0	0	0	0	-222	0	0	0	0	0	0	-222

Table S10: Energy balance [Mt CO_2/a] for carbon dioxide for the +1.7°C scenario and the three methods endogenous (*endogen*), exogenous (*exogen*) and sequential cost (*seqcost*). See also Figure S33.

budget	+1.5°C							+1.7°C							+2.0°C						
year	2020	2025	2030	2035	2040	2045	2050	2020	2025	2030	2035	2040	2045	2050	2020	2025	2030	2035	2040	2045	2050
CHP	0	0	0	0	0	0	0	-315	-1205	-1290	-1483	-1413	-1171	-288	0	-1823	-2080	-2203	-2718	-2583	-432
OCGT	-185	-13	-17	-162	-150	-131	-135	-272	-40	-67	-546	-671	-333	-139	-541	-249	-687	-1338	-1687	-1050	-153
gas boiler	-2210	-633	-427	-176	-27	0	0	-1875	-953	-691	-252	-7	0	0	-1884	-1157	-725	-295	-53	0	0
gas	4888	999	279	0	0	0	0	4930	3461	2947	2902	2523	1906	362	4920	4673	4797	4917	5457	4417	363
methanation	0	383	754	809	629	566	561	0	0	0	0	46	67	489	0	0	0	0	0	0	645
CCGT	-1971	-262	-154	-27	-3	0	0	-1946	-679	-331	-91	-13	0	0	-1973	-849	-575	-151	-21	0	0
SMR	-141	-66	-29	-22	-25	-12	-2	-141	-204	-186	-148	-83	-88	0	-141	-213	-349	-548	-597	-402	0
gas for industry	-381	-152	-152	0	0	0	0	-381	-381	-381	-381	-381	-381	0	-381	-381	-381	-381	-381	-381	0
gas for industry CC	0	-255	-255	-423	-424	-424	-424	0	0	0	0	0	0	-424	0	0	0	0	0	0	-424

Table S11: Energy balance [TWh/a] for gas for the three different budgets and the endogenous method. See also Figure S34.

method	endogen							exogen							seqcost						
year	2020	2025	2030	2035	2040	2045	2050	2020	2025	2030	2035	2040	2045	2050	2020	2025	2030	2035	2040	2045	2050
CHP	-315	-1205	-1290	-1483	-1413	-1171	-288	-115	-908	-930	-1012	-1053	-977	-141	-10	-843	-861	-1020	-1040	-974	-199
OCGT	-272	-40	-67	-546	-671	-333	-139	-289	-60	-149	-669	-873	-666	-188	-295	-79	-146	-610	-741	-481	-154
gas boiler	-1875	-953	-691	-252	-7	0	0	-1879	-880	-621	-232	-15	0	0	-2207	-945	-605	-232	-15	0	0
gas	4930	3461	2947	2902	2523	1906	362	4900	3105	2767	2671	2535	2163	363	4903	3212	2765	2877	2472	1970	363
methanation	0	0	0	0	46	67	489	0	0	0	0	0	0	390	0	0	0	0	0	9	413
CCGT	-1946	-679	-331	-91	-13	0	0	-2094	-662	-383	-90	-15	0	0	-1869	-752	-429	-101	-14	0	0
SMR	-141	-204	-186	-148	-83	-88	0	-141	-213	-303	-287	-197	-139	0	-141	-212	-343	-533	-280	-143	0
gas for industry	-381	-381	-381	-381	-381	-381	0	-381	-381	-381	-381	-381	-381	0	-381	-381	-381	-381	-381	-381	0
gas for industry CC	0	0	0	0	0	0	-424	0	0	0	0	0	0	-424	0	0	0	0	0	0	-424

Table S12: Energy balance [TWh/a] for gas for the +1.7°C scenario and the three methods endogenous (*endogen*), exogenous (*exogen*) and sequential cost (*seqcost*). See also Figure S34.

budget	+1.5°C							+1.7°C							+2.0°C						
year	2020	2025	2030	2035	2040	2045	2050	2020	2025	2030	2035	2040	2045	2050	2020	2025	2030	2035	2040	2045	2050
gas for industry	0	-229	-229	-381	-381	-381	-381	0	0	0	0	0	0	-381	0	0	0	0	0	0	-381
gas for industry CC	0	229	229	381	381	381	381	0	0	0	0	0	0	381	0	0	0	0	0	0	381

Table S13: Energy balance [TWh/a] for gas for industry for the three different budgets and the endogenous method. See also Figure S35.

method	endogen							exogen							seqcost						
year	2020	2025	2030	2035	2040	2045	2050	2020	2025	2030	2035	2040	2045	2050	2020	2025	2030	2035	2040	2045	2050
gas for industry	0	0	0	0	0	0	-381	0	0	0	0	0	0	-381	0	0	0	0	0	0	-381
gas for industry CC	0	0	0	0	0	0	381	0	0	0	0	0	0	381	0	0	0	0	0	0	381

Table S14: Energy balance [TWh/a] for gas for industry for the +1.7°C scenario and the three methods endogenous (*endogen*), exogenous (*exogen*) and sequential cost (*seqcost*). See also Figure S35.

budget	+1.5°C							+1.7°C							+2.0°C						
year	2020	2025	2030	2035	2040	2045	2050	2020	2025	2030	2035	2040	2045	2050	2020	2025	2030	2035	2040	2045	2050
BEV charger	0	184	367	596	783	781	781	0	184	369	599	783	782	782	0	183	367	597	780	782	781
Li ion	0	-183	-367	-596	-780	-780	-780	0	-183	-367	-596	-780	-780	-780	0	-183	-367	-596	-780	-780	-780
V2G	0	-1	0	0	-3	-1	-1	0	-1	-2	-3	-3	-3	-2	0	0	0	0	0	-2	-2

Table S15: Energy balance [TWh/a] for electric vehicles for the three different budgets and the endogenous method. See also Figure S36.

method	endogen							exogen							seqcost						
year	2020	2025	2030	2035	2040	2045	2050	2020	2025	2030	2035	2040	2045	2050	2020	2025	2030	2035	2040	2045	2050
BEV charger	0	184	369	599	783	782	782	0	184	368	596	781	780	783	0	184	367	596	780	780	783
Li ion	0	-183	-367	-596	-780	-780	-780	0	-183	-367	-596	-780	-780	-780	0	-183	-367	-596	-780	-780	-780
V2G	0	-1	-2	-3	-3	-3	-2	0	-1	-2	0	-1	0	-3	0	0	0	0	0	0	-3

Table S16: Energy balance [TWh/a] for electric vehicles for the +1.7°C scenario and the three methods endogenous (*endogen*), exogenous (*exogen*) and sequential cost (*seqcost*). See also Figure S36.

budget	+1.5°C							+1.7°C							+2.0°C						
year	2020	2025	2030	2035	2040	2045	2050	2020	2025	2030	2035	2040	2045	2050	2020	2025	2030	2035	2040	2045	2050
Fischer-Tropsch	0	0	0	692	1411	1592	1341	0	0	0	0	51	489	1341	0	0	0	0	0	0	1341
imported oil	5103	4344	3589	1909	377	0	0	5072	4362	3613	2606	1731	1103	0	5080	4372	3621	2601	1784	1592	0
kerosene for aviation	-612	-612	-612	-612	-612	-612	-612	-612	-612	-612	-612	-612	-612	-612	-612	-612	-612	-612	-612	-612	-612
land transport oil	-3056	-2355	-1621	-703	0	0	0	-3056	-2355	-1621	-703	0	0	0	-3056	-2355	-1621	-703	0	0	0
naphtha for industry	-728	-728	-728	-728	-728	-728	-728	-728	-728	-728	-728	-728	-728	-728	-728	-728	-728	-728	-728	-728	-728
oil boiler	-78	-20	-30	-22	-8	0	0	-46	-38	-54	-28	-1	0	0	-54	-48	-63	-23	-3	0	0
shipping oil	-629	-629	-597	-534	-440	-251	0	-629	-629	-597	-534	-440	-251	0	-629	-629	-597	-534	-440	-251	0

Table S17: Energy balance [TWh/a] for oil for the three different budgets and the endogenous method. See also Figure S37.

method	endogen							exogen							seqcost						
year	2020	2025	2030	2035	2040	2045	2050	2020	2025	2030	2035	2040	2045	2050	2020	2025	2030	2035	2040	2045	2050
Fischer-Tropsch	0	0	0	0	51	489	1341	0	0	0	0	0	138	1341	0	0	0	0	0	178	1341
imported oil	5072	4362	3613	2606	1731	1103	0	5095	4352	3613	2606	1782	1454	0	5092	4357	3609	2605	1782	1414	0
kerosene for aviation	-612	-612	-612	-612	-612	-612	-612	-612	-612	-612	-612	-612	-612	-612	-612	-612	-612	-612	-612	-612	-612
land transport oil	-3056	-2355	-1621	-703	0	0	0	-3056	-2355	-1621	-703	0	0	0	-3056	-2355	-1621	-703	0	0	0
naphtha for industry	-728	-728	-728	-728	-728	-728	-728	-728	-728	-728	-728	-728	-728	-728	-728	-728	-728	-728	-728	-728	-728
oil boiler	-46	-38	-54	-28	-1	0	0	-69	-28	-54	-27	-1	0	0	-66	-32	-50	-26	-1	0	0
shipping oil	-629	-629	-597	-534	-440	-251	0	-629	-629	-597	-534	-440	-251	0	-629	-629	-597	-534	-440	-251	0

Table S18: Energy balance [TWh/a] for oil for the +1.7°C scenario and the three methods endogenous (*endogen*), exogenous (*exogen*) and sequential cost (*seqcost*). See also Figure S37.

budget	+1.5°C							+1.7°C							+2.0°C						
year	2020	2025	2030	2035	2040	2045	2050	2020	2025	2030	2035	2040	2045	2050	2020	2025	2030	2035	2040	2045	2050
solid biomass	702	1186	1186	1186	1186	1186	1186	702	702	702	1039	1182	1122	1186	702	702	702	702	702	702	1186
CHP	0	-483	-483	-483	-405	-405	-405	0	0	0	-337	-479	-420	-405	0	0	0	0	0	0	-405
solid biomass for industry	-702	-702	-702	-702	0	0	0	-702	-702	-702	-702	-702	-702	0	-702	-702	-702	-702	-702	-702	0
solid biomass for industry CC	0	0	0	0	-780	-780	-780	0	0	0	0	0	0	-780	0	0	0	0	0	0	-780

Table S19: Energy balance [TWh/a] for solid biomass for the three different budgets and the endogenous method. See also Figure S38.

method	endogen							exogen							seqcost						
year	2020	2025	2030	2035	2040	2045	2050	2020	2025	2030	2035	2040	2045	2050	2020	2025	2030	2035	2040	2045	2050
solid biomass	702	702	702	1039	1182	1122	1186	702	929	1097	1186	1186	1186	1186	702	1010	1184	1186	1186	1186	1186
CHP	0	0	0	-337	-479	-420	-405	0	-227	-395	-483	-483	-483	-405	0	-308	-481	-483	-483	-483	-405
solid biomass for industry	-702	-702	-702	-702	-702	-702	0	-702	-702	-702	-702	-702	-702	0	-702	-702	-702	-702	-702	-702	0
solid biomass for industry CC	0	0	0	0	0	0	-780	0	0	0	0	0	0	-780	0	0	0	0	0	0	-780

Table S20: Energy balance [TWh/a] for solid biomass for industry for the +1.7°C scenario and the three methods endogenous (*endogen*), exogenous (*exogen*) and sequential cost (*seqcost*). See also Figure S38.

budget	+1.5°C							+1.7°C							+2.0°C						
year	2020	2025	2030	2035	2040	2045	2050	2020	2025	2030	2035	2040	2045	2050	2020	2025	2030	2035	2040	2045	2050
solid biomass for industry	0	0	0	0	-702	-702	-702	0	0	0	0	0	0	-702	0	0	0	0	0	0	-702
solid biomass for industry CC	0	0	0	0	702	702	702	0	0	0	0	0	0	702	0	0	0	0	0	0	702

Table S21: Energy balance [TWh/a] for solid biomass for industry for the three different budgets and the endogenous method. See also Figure S39.

method	endogen							exogen							seqcost						
year	2020	2025	2030	2035	2040	2045	2050	2020	2025	2030	2035	2040	2045	2050	2020	2025	2030	2035	2040	2045	2050
solid biomass for industry	0	0	0	0	0	0	-702	0	0	0	0	0	0	-702	0	0	0	0	0	0	-702
solid biomass for industry CC	0	0	0	0	0	0	702	0	0	0	0	0	0	702	0	0	0	0	0	0	702

Table S22: Energy balance [TWh/a] for solid biomass for the +1.7°C scenario and the three methods endogenous (*endogen*), exogenous (*exogen*) and sequential cost (*seqcost*). See also Figure S39.

budget	+1.5°C							+1.7°C							+2.0°C						
year	2020	2025	2030	2035	2040	2045	2050	2020	2025	2030	2035	2040	2045	2050	2020	2025	2030	2035	2040	2045	2050
process emissions	0	166	166	166	162	157	153	0	0	0	0	24	153	153	0	0	0	0	0	0	153
process emissions CC	0	-166	-166	-166	-162	-157	-153	0	0	0	0	-24	-153	-153	0	0	0	0	0	0	-153

Table S23: Energy balance [TWh/a] for process emissions for the three different budgets and the endogenous method. See also Figure S40.

method	endogen							exogen							seqcost						
year	2020	2025	2030	2035	2040	2045	2050	2020	2025	2030	2035	2040	2045	2050	2020	2025	2030	2035	2040	2045	2050
process emissions	0	0	0	0	24	153	153	0	0	0	0	0	39	153	0	0	0	0	0	52	153
process emissions CC	0	0	0	0	-24	-153	-153	0	0	0	0	0	-39	-153	0	0	0	0	0	-52	-153

Table S24: Energy balance [TWh/a] for process emissions for the +1.7°C scenario and the three methods endogenous (*endogen*), exogenous (*exogen*) and sequential cost (*seqcost*). See also Figure S40.

budget	+1.5°C							+1.7°C							+2.0°C						
year	2020	2025	2030	2035	2040	2045	2050	2020	2025	2030	2035	2040	2045	2050	2020	2025	2030	2035	2040	2045	2050
CHP	0	0	0	0	0	1	109	0	0	0	0	0	0	0	0	0	0	0	0	0	115
methanation	0	-77	-151	-162	-126	-113	-112	0	0	0	0	-9	-13	-98	0	0	0	0	0	0	-129
CO2 sequestration	0	-119	-44	123	40	0	-200	0	0	0	0	0	0	-200	0	0	0	0	0	0	-200
DAC	0	0	0	0	22	102	118	0	0	0	0	0	0	212	0	0	0	0	0	0	127
Fischer-Tropsch	0	0	0	-187	-381	-430	-362	0	0	0	0	-14	-132	-362	0	0	0	0	0	0	-362
gas for industry CC	0	46	46	77	77	77	79	0	0	0	0	0	0	80	0	0	0	0	0	0	80
process emissions CC	0	149	149	149	145	141	145	0	0	0	0	23	145	145	0	0	0	0	0	0	145
solid biomass for industry CC	0	0	0	0	222	222	222	0	0	0	0	0	0	222	0	0	0	0	0	0	222

Table S25: Energy balance [Mt CO_2/a] for carbon dioxide storage for the three different budgets and the endogenous method. See also Figure S41.

method	endogen							exogen							seqcost						
year	2020	2025	2030	2035	2040	2045	2050	2020	2025	2030	2035	2040	2045	2050	2020	2025	2030	2035	2040	2045	2050
CHP	0	0	0	0	0	0	0	0	0	0	0	0	0	21	0	0	0	0	0	0	27
methanation	0	0	0	0	-9	-13	-98	0	0	0	0	0	0	-78	0	0	0	0	0	-2	-83
CO2 sequestration	0	0	0	0	0	0	-200	0	0	0	0	0	0	-200	0	0	0	0	0	0	-200
DAC	0	0	0	0	0	0	212	0	0	0	0	0	0	171	0	0	0	0	0	0	170
Fischer-Tropsch	0	0	0	0	-14	-132	-362	0	0	0	0	0	-37	-362	0	0	0	0	0	-48	-362
gas for industry CC	0	0	0	0	0	0	80	0	0	0	0	0	0	80	0	0	0	0	0	0	80
process emissions CC	0	0	0	0	23	145	145	0	0	0	0	0	37	145	0	0	0	0	0	50	145
solid biomass for industry CC	0	0	0	0	0	0	222	0	0	0	0	0	0	222	0	0	0	0	0	0	222

Table S26: Energy balance [Mt CO_2/a] for carbon dioxide storage for the +1.7°C scenario and the three methods endogenous (*endogen*), exogenous (*exogen*) and sequential cost (*seqcost*). See also Figure S41.

budget	+1.5°C							+1.7°C							+2.0°C						
year	2020	2025	2030	2035	2040	2045	2050	2020	2025	2030	2035	2040	2045	2050	2020	2025	2030	2035	2040	2045	2050
ground heat pump	64	401	479	417	257	218	153	64	187	325	414	257	218	153	80	187	288	418	257	218	173
resistive heater	0	105	77	280	485	490	500	0	65	87	267	485	490	500	0	20	48	249	485	490	480
gas boiler	721	285	226	72	0	0	0	721	533	349	83	0	0	0	705	578	415	99	0	0	0
heat	-785	-791	-791	-776	-742	-708	-653	-785	-791	-791	-776	-742	-708	-653	-785	-791	-791	-776	-742	-708	-653
oil boiler	0	0	8	7	0	0	0	0	6	30	12	0	0	0	0	6	40	9	0	0	0

Table S27: Energy balance [TWh/a] for rural heat for the three different budgets and the endogenous method. See also Figure S42.

method	endogen							exogen							seqcost						
year	2020	2025	2030	2035	2040	2045	2050	2020	2025	2030	2035	2040	2045	2050	2020	2025	2030	2035	2040	2045	2050
ground heat pump	64	187	325	414	257	218	153	65	220	366	414	257	218	154	64	225	381	413	257	218	154
resistive heater	0	65	87	267	485	490	500	0	80	78	267	485	490	500	0	40	74	267	485	490	500
gas boiler	721	533	349	83	0	0	0	720	487	320	83	0	0	0	721	522	310	83	0	0	0
heat	-785	-791	-791	-776	-742	-708	-653	-785	-791	-791	-776	-742	-708	-653	-785	-791	-791	-776	-742	-708	-653
oil boiler	0	6	30	12	0	0	0	0	4	28	12	0	0	0	0	4	26	13	0	0	0

Table S28: Energy balance [TWh/a] for rural heat for the +1.7°C scenario and the three methods endogenous (*endogen*), exogenous (*exogen*) and sequential cost (*seqcost*). See also Figure S42.

budget	+1.5°C							+1.7°C							+2.0°C						
year	2020	2025	2030	2035	2040	2045	2050	2020	2025	2030	2035	2040	2045	2050	2020	2025	2030	2035	2040	2045	2050
air heat pump	1011	1683	1667	1562	1499	1245	974	993	1342	1375	1289	1493	1199	1093	895	793	621	536	754	755	865
resistive heater	6	19	1	4	0	0	0	217	227	207	202	0	0	0	337	604	878	922	657	393	119
gas boiler	1054	259	127	60	0	0	0	885	380	208	117	0	0	0	857	544	288	129	0	0	0
hot water storage	0	0	-5	-36	-107	-18	-3	0	0	-1	-21	-128	-105	0	0	0	0	0	-46	-55	0
DAC	0	0	0	0	-28	-133	-153	0	0	0	0	0	0	-275	0	0	0	0	0	0	-166
heat	-2136	-1978	-1804	-1597	-1365	-1094	-818	-2136	-1978	-1804	-1597	-1365	-1094	-818	-2136	-1978	-1804	-1597	-1365	-1094	-818
oil boiler	65	17	14	7	0	0	0	42	28	15	11	0	0	0	47	37	17	10	0	0	0

Table S29: Energy balance [TWh/a] for urban individual heat for the three different budgets and the endogenous method. See also Figure S43.

method	endogen							exogen							seqcost						
year	2020	2025	2030	2035	2040	2045	2050	2020	2025	2030	2035	2040	2045	2050	2020	2025	2030	2035	2040	2045	2050
air heat pump	993	1342	1375	1289	1493	1199	1093	1232	1592	1565	1524	1493	1199	1041	1021	1566	1566	1513	1493	1199	1040
resistive heater	217	227	207	202	0	0	0	6	17	2	1	0	0	0	6	14	3	2	0	0	0
gas boiler	885	380	208	117	0	0	0	837	349	222	99	0	0	0	1054	374	222	111	0	0	0
hot water storage	0	0	-1	-21	-128	-105	0	0	0	-4	-36	-128	-105	0	0	0	-3	-36	-128	-105	-1
DAC	0	0	0	0	0	0	-275	0	0	0	0	0	0	-223	0	0	0	0	0	0	-220
heat	-2136	-1978	-1804	-1597	-1365	-1094	-818	-2136	-1978	-1804	-1597	-1365	-1094	-818	-2136	-1978	-1804	-1597	-1365	-1094	-818
oil boiler	42	28	15	11	0	0	0	61	21	18	10	0	0	0	56	25	17	8	0	0	0

Table S30: Energy balance [TWh/a] for urban individual heat for the +1.7°C scenario and the three methods endogenous (*endogen*), exogenous (*exogen*) and sequential cost (*seqcost*). See also Figure S43.

budget	+1.5°C							+1.7°C							+2.0°C						
year	2020	2025	2030	2035	2040	2045	2050	2020	2025	2030	2035	2040	2045	2050	2020	2025	2030	2035	2040	2045	2050
air heat pump	25	181	421	505	532	754	813	64	118	146	111	211	436	713	139	1	39	47	0	102	552
resistive heater	0	36	21	2	0	0	0	0	1	28	12	0	0	0	0	0	0	8	0	148	138
CHP	0	333	333	333	280	280	298	131	495	529	844	913	771	400	0	744	849	900	1118	1061	481
gas boiler	392	74	64	41	28	0	0	226	12	121	48	7	0	0	281	0	0	62	54	0	0
hot water storage	0	0	-18	-101	-206	-262	-253	0	0	-1	-9	-18	-59	-193	0	-120	-62	-9	-20	-24	-208
Fischer-Tropsch	0	0	0	221	437	491	411	0	0	0	0	15	139	368	0	0	0	0	0	0	358
H2 Fuel Cell	0	0	0	0	78	83	200	0	0	0	0	25	59	181	0	0	0	0	0	58	149
heat	-422	-626	-826	-1009	-1156	-1346	-1470	-422	-626	-826	-1009	-1156	-1346	-1470	-422	-626	-826	-1009	-1156	-1346	-1470
oil boiler	5	1	5	6	7	0	0	0	0	3	2	1	0	0	1	0	0	1	3	0	0

Table S31: Energy balance [TWh/a] for urban district heat for the three different budgets and the endogenous method. See also Figure S44.

method	endogen							exogen							seqcost						
year	2020	2025	2030	2035	2040	2045	2050	2020	2025	2030	2035	2040	2045	2050	2020	2025	2030	2035	2040	2045	2050
air heat pump	64	118	146	111	211	436	713	91	93	105	210	380	580	825	25	70	83	217	391	566	793
resistive heater	0	1	28	12	0	0	0	0	4	24	8	0	0	0	0	4	19	11	0	7	41
CHP	131	495	529	844	913	771	400	48	528	653	749	766	735	343	4	557	684	753	761	734	368
gas boiler	226	12	121	48	7	0	0	282	20	64	46	15	0	0	389	23	58	33	16	0	0
hot water storage	0	0	-1	-9	-18	-59	-193	0	-18	-22	-7	-15	-16	-190	0	-29	-21	-6	-17	-13	-185
Fischer-Tropsch	0	0	0	0	15	139	368	0	0	0	0	0	39	360	0	0	0	0	0	50	361
H2 Fuel Cell	0	0	0	0	25	59	181	0	0	0	0	7	7	132	0	0	0	0	3	3	92
heat	-422	-626	-826	-1009	-1156	-1346	-1470	-422	-626	-826	-1009	-1156	-1346	-1470	-422	-626	-826	-1009	-1156	-1346	-1470
oil boiler	0	0	3	2	1	0	0	2	0	3	2	1	0	0	4	0	3	2	1	0	0

Table S32: Energy balance [TWh/a] for urban district heat for the +1.7°C scenario and the three methods endogenous (*endogen*), exogenous (*exogen*) and sequential cost (*seqcost*). See also Figure S44.

budget	+1.5°C							+1.7°C							+2.0°C						
year	2020	2025	2030	2035	2040	2045	2050	2020	2025	2030	2035	2040	2045	2050	2020	2025	2030	2035	2040	2045	2050
nuclear	-2328	-2547	-2664	-2672	-2712	-2379	-352	-3105	-2975	-2609	-2643	-2601	-2166	-350	-3105	-3105	-3028	-2807	-2677	-2313	-350
uranium	2328	2547	2664	2672	2712	2379	352	3105	2975	2609	2643	2601	2166	350	3105	3105	3028	2807	2677	2313	350

Table S33: Energy balance [TWh/a] for uranium for the three different budgets and the endogenous method. See also Figure S45.

method	endogen							exogen							seqcost						
year	2020	2025	2030	2035	2040	2045	2050	2020	2025	2030	2035	2040	2045	2050	2020	2025	2030	2035	2040	2045	2050
nuclear	-3105	-2975	-2609	-2643	-2601	-2166	-350	-3105	-2780	-2650	-2572	-2463	-2261	-350	-3030	-3053	-2695	-2345	-2371	-2266	-344
uranium	3105	2975	2609	2643	2601	2166	350	3105	2780	2650	2572	2463	2261	350	3030	3053	2695	2345	2371	2266	344

Table S34: Energy balance [TWh/a] for uranium for the +1.7°C scenario and the three methods endogenous (*endogen*), exogenous (*exogen*) and sequential cost (*seqcost*). See also Figure S45.

method	endogen							seqcost							exogen						
year	2020	2025	2030	2035	2040	2045	2050	2020	2025	2030	2035	2040	2045	2050	2020	2025	2030	2035	2040	2045	2050
Offshore wind [GW]	35	187	193	193	190	182	175	41	162	184	316	506	498	478	18	65	155	207	255	250	263
Solar rooftop [GW]	0	0	0	0	16	16	16	0	58	96	96	96	96	97	0	0	5	80	96	96	96
Solar PV [GW]	172	436	940	1970	2616	3106	3335	153	337	516	1488	1509	1818	2611	153	577	791	1168	2217	2203	2419
Onshore wind [GW]	257	1031	1222	1530	1811	1774	1785	199	715	1235	1315	1671	1634	1599	199	986	1240	1540	1958	1921	1890
OCGT [GW]	96	60	55	371	449	429	444	155	120	107	331	363	328	405	171	136	123	379	470	427	464
CCGT [GW]	241	153	103	24	4	0	0	241	153	103	24	4	0	0	241	153	103	24	4	0	0
Hydroelectricity [GW]	188	188	188	188	188	188	188	188	188	188	188	188	188	188	188	188	188	188	188	188	188
Nuclear [GW]	354	354	354	354	341	299	44	354	354	354	354	341	299	44	354	354	354	354	341	299	44
SMR CC [GW]	0	0	0	0	0	0	0	0	0	0	0	0	0	0	0	0	0	0	0	0	0
OCGT H2 [GW]	0	0	0	0	0	0	0	0	0	0	0	0	0	0	0	0	0	0	0	0	0
H2 boiler [GW]	0	0	0	0	0	0	0	0	0	0	0	0	0	0	0	0	0	0	0	0	0
H2 Fuel Cell [GW]	0	0	0	0	123	151	258	0	0	0	0	16	62	170	0	0	0	0	25	81	196
Solar thermal [GW]	0	0	0	0	0	0	0	0	0	0	0	0	0	0	0	0	0	0	0	0	80
DAC [GW]	0	0	0	0	2	12	15	0	0	0	0	0	5	15	0	0	0	0	0	0	7
Fischer-Tropsch [GW]	0	0	0	137	276	299	299	0	0	0	152	337	337	337	0	0	0	81	337	337	337
SMR [GW]	16	17	17	17	17	17	1	16	25	25	25	25	25	8	16	21	21	21	21	21	5
Ground heat pump [GW]	2	18	23	23	22	18	11	7	21	28	27	22	18	11	8	22	29	28	22	18	11
Gas for industry CC [GW]	0	29	29	48	48	48	48	0	0	0	14	48	48	48	0	0	0	0	48	48	48
Gas for industry [GW]	43	43	43	43	43	43	43	43	43	43	43	43	43	43	43	43	43	43	43	43	43
Resistive heater [GW]	219	161	104	144	199	199	199	219	161	104	127	199	210	218	219	161	104	124	199	199	199
CHP [GW]	0	119	126	126	126	126	102	0	125	133	133	133	133	96	0	121	130	130	130	130	101
Oil boiler [GW]	324	242	159	76	8	0	0	324	242	159	76	8	0	0	324	242	159	76	8	0	0
Methanation [GW]	0	119	232	280	280	280	280	0	0	154	154	154	154	154	16	20	126	127	127	127	110
Air heat pump [GW]	149	181	239	268	272	257	214	149	181	239	265	285	264	221	149	181	240	272	283	267	222
Gas boiler [GW]	680	506	333	159	16	0	0	680	506	333	159	16	0	0	680	506	333	159	16	0	0
H2 Electrolysis [GW]	18	214	435	1031	1377	1667	2186	20	20	304	659	1155	1324	1818	0	57	249	434	1093	1152	1472
Electricity distribution grid [GW]	539	628	718	877	995	995	995	543	612	704	858	989	989	989	543	634	724	863	982	982	982
Battery storage [TWh]	0	0	0	0	0	0	0	0	0	0	0	0	0	0	0	0	0	0	0	0	0
Hot water storage [TWh]	1	1	2	3	3	3	3	1	1	1	2	2	2	2	1	1	1	2	2	2	2
Li ion [TWh]	0	1	2	4	5	5	5	0	1	2	4	5	5	5	0	1	2	4	5	5	5
BEV charger [TW]	0	5	11	18	23	23	23	0	5	11	18	23	23	23	0	5	11	18	23	23	23
V2G [TW]	0	5	11	18	23	23	23	0	5	11	18	23	23	23	0	5	11	18	23	23	23
H2 Store [TWh]	0	4	12	48	158	235	349	0	1	20	59	196	196	297	0	2	13	41	188	188	299
CO2 sequestration [Mt_{CO_2}/a]	0	118	180	200	118	80	200	0	138	187	199	116	83	200	0	120	170	194	165	101	200

Table S35: Installed capacities in the +1.5°C scenarios.

method	endogen							seqcost							exogen						
year	2020	2025	2030	2035	2040	2045	2050	2020	2025	2030	2035	2040	2045	2050	2020	2025	2030	2035	2040	2045	2050
Offshore wind [GW]	27	82	97	96	94	86	190	41	74	96	118	138	230	367	18	41	41	47	108	171	257
Solar rooftop [GW]	0	0	0	0	16	16	56	0	0	96	96	96	96	96	0	0	1	33	96	96	99
Solar PV [GW]	172	423	764	972	1455	2165	3152	153	310	379	559	774	1118	2550	153	347	640	811	997	1197	2707
Onshore wind [GW]	218	572	678	728	761	1016	1702	181	557	637	748	961	924	1651	184	661	693	835	882	923	1802
OCGT [GW]	142	106	94	315	489	422	422	152	117	104	393	626	549	549	149	114	101	397	614	540	540
CCGT [GW]	241	153	103	24	4	0	0	241	153	103	24	4	0	0	241	153	103	24	4	0	0
Hydroelectricity [GW]	188	188	188	188	188	188	188	188	188	188	188	188	188	188	188	188	188	188	188	188	188
Nuclear [GW]	354	354	354	354	341	299	44	354	354	354	354	341	299	44	354	354	354	354	341	299	44
SMR CC [GW]	0	0	0	0	0	0	0	0	0	0	0	0	0	0	0	0	0	0	0	0	0
OCGT H2 [GW]	0	0	0	0	0	0	0	0	0	0	0	42	42	42	0	0	0	0	0	0	0
H2 boiler [GW]	0	0	0	0	0	0	0	0	0	0	0	0	0	0	0	0	0	0	0	0	0
H2 Fuel Cell [GW]	0	0	0	0	24	101	229	0	0	0	0	20	20	134	0	0	0	0	27	50	176
Solar thermal [GW]	0	0	0	0	0	0	0	0	0	0	0	0	0	0	0	0	0	0	0	0	0
DAC [GW]	0	0	0	0	0	0	27	0	0	0	0	0	0	21	0	0	0	0	0	0	22
Fischer-Tropsch [GW]	0	0	0	0	10	107	249	0	0	0	0	0	47	257	0	0	0	0	0	38	260
SMR [GW]	16	28	37	37	37	37	21	16	29	45	68	68	68	51	16	30	45	63	63	63	47
Ground heat pump [GW]	2	6	13	23	22	18	11	2	7	16	23	22	18	11	2	7	15	23	22	18	11
Gas for industry CC [GW]	0	0	0	0	0	0	48	0	0	0	0	0	0	48	0	0	0	0	0	0	48
Gas for industry [GW]	43	43	43	43	43	43	43	43	43	43	43	43	43	43	43	43	43	43	43	43	43
Resistive heater [GW]	304	247	189	230	199	199	199	219	161	104	144	199	202	214	219	161	104	144	199	199	199
CHP [GW]	92	375	375	542	596	504	220	3	304	340	444	466	463	184	26	319	349	450	467	441	166
Oil boiler [GW]	324	242	159	76	8	0	0	324	242	159	76	8	0	0	324	242	159	76	8	0	0
Methanation [GW]	0	0	0	0	18	29	273	0	0	0	0	0	3	161	0	0	0	0	0	0	128
Air heat pump [GW]	116	148	176	182	230	216	203	149	181	206	227	248	236	220	149	181	208	225	246	237	220
Gas boiler [GW]	680	506	333	159	16	0	0	680	506	333	159	16	0	0	680	506	333	159	16	0	0
H2 Electrolysis [GW]	0	9	60	149	406	697	1733	0	1	3	8	112	335	1389	0	0	16	74	173	332	1384
Electricity distribution grid [GW]	581	643	715	863	951	951	951	539	609	663	814	953	953	953	539	613	676	822	952	952	952
Battery storage [TWh]	0	0	0	0	0	0	0	0	0	0	0	0	0	0	0	0	0	0	0	0	0
Hot water storage [TWh]	1	1	1	2	3	3	3	0	0	0	2	5	5	5	0	0	0	2	5	5	5
Li ion [TWh]	0	1	2	4	5	5	5	0	1	2	4	5	5	5	0	1	2	4	5	5	5
BEV charger [TW]	0	5	11	18	23	23	23	0	5	11	18	23	23	23	0	5	11	18	23	23	23
V2G [TW]	0	5	11	18	23	23	23	0	5	11	18	23	23	23	0	5	11	18	23	23	23
H2 Store [TWh]	0	0	2	16	59	102	312	0	0	1	5	23	70	261	0	0	1	4	46	85	291
CO2 sequestration [Mt_{CO_2}/a]	0	0	0	0	5	26	200	0	0	0	0	0	10	200	0	0	0	0	0	7	200

Table S36: Installed capacities in the +1.7°C scenarios.

method	endogen							seqcost							exogen						
year	2020	2025	2030	2035	2040	2045	2050	2020	2025	2030	2035	2040	2045	2050	2020	2025	2030	2035	2040	2045	2050
Offshore wind [GW]	18	22	22	21	20	30	190	18	18	18	18	15	30	204	18	18	18	18	15	10	245
Solar rooftop [GW]	0	0	0	0	16	16	17	0	0	62	62	97	99	218	0	0	0	25	81	81	99
Solar PV [GW]	167	345	422	747	879	1602	3178	153	153	177	540	589	579	2054	153	153	472	558	709	810	2852
Onshore wind [GW]	263	427	555	617	609	580	1839	277	373	513	570	586	644	2251	276	373	488	608	561	615	1833
OCGT [GW]	320	285	272	668	662	519	519	325	289	277	464	634	629	629	325	289	277	578	622	581	581
CCGT [GW]	241	153	103	24	4	0	0	241	153	103	24	4	0	0	241	153	103	24	4	0	0
Hydroelectricity [GW]	188	188	188	188	188	188	188	188	188	188	188	188	188	188	188	188	188	188	188	188	188
Nuclear [GW]	354	354	354	354	341	299	44	354	354	354	354	341	299	44	354	354	354	354	341	299	44
SMR CC [GW]	0	0	0	0	0	0	0	0	0	0	0	0	0	0	0	0	0	0	0	0	0
OCGT H2 [GW]	0	0	0	0	0	0	0	0	0	0	0	0	133	133	0	0	0	0	0	0	0
H2 boiler [GW]	0	0	0	0	0	0	0	0	0	0	0	0	0	0	0	0	0	0	0	0	0
H2 Fuel Cell [GW]	0	0	0	0	0	41	193	0	0	0	0	12	17	104	0	0	0	0	0	13	195
Solar thermal [GW]	0	0	0	0	0	0	0	0	0	0	0	0	0	0	0	0	0	0	0	0	0
DAC [GW]	0	0	0	0	0	0	16	0	0	0	0	0	0	6	0	0	0	0	0	0	13
Fischer-Tropsch [GW]	0	0	0	0	0	0	245	0	0	0	0	0	0	279	0	0	0	0	0	0	244
SMR [GW]	16	29	47	70	72	72	56	16	30	51	80	103	116	100	16	30	51	74	91	104	87
Ground heat pump [GW]	2	6	11	23	22	18	13	2	6	8	23	22	18	16	2	6	9	23	22	18	15
Gas for industry CC [GW]	0	0	0	0	0	0	48	0	0	0	0	0	0	48	0	0	0	0	0	0	48
Gas for industry [GW]	43	43	43	43	43	43	43	43	43	43	43	43	43	43	43	43	43	43	43	43	43
Resistive heater [GW]	355	410	475	531	475	410	287	364	306	371	396	306	320	197	363	361	433	459	370	361	231
CHP [GW]	0	685	685	691	835	835	246	0	757	757	757	757	757	95	0	713	713	713	824	824	206
Oil boiler [GW]	324	242	159	76	8	0	0	324	242	159	76	8	0	0	324	242	159	76	8	0	0
Methanation [GW]	0	0	0	0	0	0	318	0	0	0	0	0	0	73	0	0	0	0	0	0	158
Air heat pump [GW]	96	84	72	60	104	111	164	93	125	116	113	179	163	236	93	103	91	88	147	136	190
Gas boiler [GW]	680	506	333	159	16	0	0	680	506	333	159	16	0	0	680	506	333	159	16	0	0
H2 Electrolysis [GW]	0	1	3	9	62	490	1664	0	0	0	0	0	0	1065	0	0	0	4	10	42	1378
Electricity distribution grid [GW]	606	721	855	1012	1072	1072	1072	610	670	800	927	983	983	983	610	698	836	966	1009	1009	1009
Battery storage [TWh]	0	0	0	0	0	0	0	0	0	0	0	0	0	0	0	0	0	0	0	0	0
Hot water storage [TWh]	1	1	1	1	2	3	3	0	0	0	0	3	4	4	1	1	1	1	2	3	3
Li ion [TWh]	0	1	2	4	5	5	5	0	1	2	4	5	5	5	0	1	2	4	5	5	5
BEV charger [TW]	0	5	11	18	23	23	23	0	5	11	18	23	23	23	0	5	11	18	23	23	23
V2G [TW]	0	5	11	18	23	23	23	0	5	11	18	23	23	23	0	5	11	18	23	23	23
H2 Store [TWh]	0	0	1	3	4	50	278	0	0	1	3	5	13	244	0	0	1	3	6	6	294
CO2 sequestration [Mt_{CO_2}/a]	0	0	0	0	0	0	200	0	0	0	0	0	0	200	0	0	0	0	0	0	200

Table S37: Installed capacities in the +2.0°C scenarios.

Supplementary References

[1] International Renewable Energy Agency: Global hydrogen trade to meet the 1.5°C climate goal: Part III – Green hydrogen cost and potential (2022). https://www.irena.org/publications/2022/May/Global-hydrogen-trade-Cost

[2] Energy Transitions Commission: Making the Hydrogen Economy Possible: Accelerating Clean Hydrogen in an Electrified Economy (2021). https://www.energy-transitions.org/publications/making-clean-hydrogen-possible/#download-form

[3] International Energy Agency (IEA): Global Hydrogen Review 2022 (2022). https://iea.blob.core.windows.net/assets/c5bc75b1-9e4d-460d-9056-6e8e626a11c4/GlobalHydrogenReview2022.pdf

[4] Vartiainen, E., Breyer, C., Moser, D., Román Medina, E., Busto, C., Masson, G., Bosch, E., Jäger-Waldau, A.: True cost of solar hydrogen. Solar RRL **6**(5), 2100487 (2022) https://arxiv.org/abs/https://onlinelibrary.wiley.com/doi/pdf/10.1002/solr.202100487. https://doi.org/10.1002/solr.202100487

[5] R. Ortiz Cebolla, F. Dolci, E. Weidner Ronnefeld: Assessment of hydrogen delivery options (KJ-NA-31-199-EN-N (online)) (2022). https://doi.org/10.2760/869085

[6] Hydrogen Council: Hydrogen decarbonization pathways (2021). https://hydrogencouncil.com/wp-content/uploads/2021/01/Hydrogen-Council-Report_Decarbonization-Pathways_Part-2_Supply-Scenarios.pdf

[7] BloombergNEF: Hydrogen Economy Outlook (2020). https://data.bloomberglp.com/professional/sites/24/BNEF-Hydrogen-Economy-Outlook-Key-Messages-30-Mar-2020.pdf

[8] Fraunhofer-Institute for solar energy systems (ISE): Cost forecast for low temperature electrolysis - technology driven bottom-up prognosis for PEM and alkaline water electrolysis systems (2021). https://www.ise.fraunhofer.de/content/dam/ise/de/documents/publications/studies/cost-forecast-for-low-temperature-electrolysis.pdf

[9] Agency, I.E.: The Future of Hydrogen, p. 203 (2019). https://doi.org/10.1787/1e0514c4-en. https://doi.org/https://doi.org/10.1787/1e0514c4-en

[10] IRENA: Green Hydrogen Cost Reduction: Scaling up Electrolysers to Meet the 1.5^{o}C Climate Goal. Abu Dhabi (2020). https://www.irena.org/-/media/Files/IRENA/Agency/Publication/2020/Dec/IRENA_Green_hydrogen_cost_2020.pdf

[11] Odenweller, A., Ueckerdt, F., Nemet, G.F., Jensterle, M., Luderer, G.: Probabilistic feasibility space of scaling up green hydrogen supply. Nature Energy

7(9), 854–865 (2022). https://doi.org/10.1038/s41560-022-01097-4

[12] Seck, G.S., Hache, E., Sabathier, J., Guedes, F., Reigstad, G.A., Straus, J., Wolfgang, O., Ouassou, J.A., Askeland, M., Hjorth, I., Skjelbred, H.I., Andersson, L.E., Douguet, S., Villavicencio, M., Trüby, J., Brauer, J., Cabot, C.: Hydrogen and the decarbonization of the energy system in europe in 2050: A detailed model-based analysis. Renewable and Sustainable Energy Reviews **167**, 112779 (2022). https://doi.org/10.1016/j.rser.2022.112779

[13] Way, R., Ives, M.C., Mealy, P., Farmer, J.D.: Empirically grounded technology forecasts and the energy transition. Joule **6**(9), 2057–2082 (2022). https://doi.org/10.1016/j.joule.2022.08.009

[14] Egli, F., Steffen, B., Schmidt, T.: A dynamic analysis of financing conditions for renewable energy technologies. Nature Energy **3** (2018). https://doi.org/10.1038/s41560-018-0277-y

[15] Victoria, M., Zeyen, E., Brown, T.: Speed of technological transformations required in Europe to achieve different climate goals (2022). https://doi.org/10.1016/j.joule.2022.04.016. https://doi.org/10.1016/j.joule.2022.04.016

[16] IEA: Renewables 2020 Data Explorer (2020). https://www.iea.org/articles/renewables-2020-data-explorer?mode=market&region=Germany&product=PV

[17] European Commission, Joint Research Centre (JRC): JRC-IDEES 2015. Mantzos, Leonidas; Matei, Nicoleta Anca; Mulholland, Eamonn; Rózsai, Máté; Tamba, Marie; Wiesenthal, Tobias (2018). https://doi.org/10.2905/JRC-10110-10001. https://doi.org/10.2905/JRC-10110-10001

[18] Fraunhofer ISE: Wege zu einem klimaneutralen Energiesystem (2020). https://www.ise.fraunhofer.de/de/veroeffentlichungen/studien/wege-zu-einem-klimaneutralen-energiesystem.html

[19] BloombergNEF: Electric Vehicle Outlook 2021 (2021). https://about.bnef.com/electric-vehicle-outlook/

[20] European Commission: European CO2 storage database (2011). https://setis.ec.europa.eu/european-co2-storage-database_en

[21] Danish Energy Agency (DEA): Technology Data (2022). https://ens.dk/en/our-services/projections-and-models/technology-data

[22] Leigh Collins: Record breaker - World's largest green hydrogen project, with 150MW electrolyser, brought on line in China . Recharge. Accessed on 3.11.22 (2022). https://www.rechargenews.com/energy-transition/record-breaker-world-s-largest-green-hydrogen-project-with-150mw-electrolyser-brought-on-line-in-china/2-1-1160799

[23] Everfuel is leading the flagship project, HySynergy, in establishing a 20 MW PtX facility. . Everfuel. Accessed on 3.11.22 (2022). https://www.everfuel.com/projects/hysynergy/

[24] Reksten, A.H., Thomassen, M.S., Møller-Holst, S., Sundseth, K.: Projecting the future cost of pem and alkaline water electrolysers; a capex model including electrolyser plant size and technology development. International Journal of Hydrogen Energy **47**(90), 38106–38113 (2022). https://doi.org/10.1016/j.ijhydene.2022.08.306

[25] Schmidt, O., Gambhir, A., Staffell, I., Hawkes, A., Nelson, J., Few, S.: Future cost and performance of water electrolysis: An expert elicitation study. International Journal of Hydrogen Energy **42**(52), 30470–30492 (2017). https://doi.org/10.1016/j.ijhydene.2017.10.045

[26] Wei, M., Smith, S.J., Sohn, M.D.: Experience curve development and cost reduction disaggregation for fuel cell markets in japan and the us. Applied Energy **191**, 346–357 (2017). https://doi.org/10.1016/j.apenergy.2017.01.056

[27] Böhm, H., Goers, S., Zauner, A.: Estimating future costs of power-to-gas – a component-based approach for technological learning. International Journal of Hydrogen Energy **44**(59), 30789–30805 (2019). https://doi.org/10.1016/j.ijhydene.2019.09.230

[28] Rivera-Tinoco, R., Schoots, K., van der Zwaan, B.: Learning curves for solid oxide fuel cells. Energy Conversion and Management **57**, 86–96 (2012). https://doi.org/10.1016/j.enconman.2011.11.018

[29] Böhm, H., Zauner, A., Rosenfeld, D.C., Tichler, R.: Projecting cost development for future large-scale power-to-gas implementations by scaling effects. Applied Energy **264**, 114780 (2020). https://doi.org/10.1016/j.apenergy.2020.114780

[30] Frysztacki, M.M., Hörsch, J., Hagenmeyer, V., Brown, T.: The strong effect of network resolution on electricity system models with high shares of wind and solar. Applied Energy **291**, 116726 (2021). https://doi.org/10.1016/j.apenergy.2021.116726

[31] F. Neumann, E. Zeyen, M. Victoria, T. Brown: The Potential Role of a Hydrogen Network in Europe (2023) https://arxiv.org/abs/2207.05816 [physics.soc-ph]. https://doi.org/10.48550/arXiv.2207.05816

[32] Github repository Technology Data. https://github.com/PyPSA/technology-data

[33] Lauri, K., Jouko, R., Nicklas, N., Sebastian, T.: Scenarios and new technologies for a north-european co2 transport infrastructure in 2050. Energy Procedia

63, 2738–2756 (2014). https://doi.org/10.1016/j.egypro.2014.11.297. 12th International Conference on Greenhouse Gas Control Technologies, GHGT-12

[34] Lazard's Levelized Cost of Energy ("LCOE") analysis - Version 13.0 . Lazard (2019). https://www.lazard.com/media/451086/lazards-levelized-cost-of-energy-version-130-vf.pdf

[35] Fasihi, M., Bogdanov, D., Breyer, C.: Long-term hydrocarbon trade options for the maghreb region and europe—renewable energy based synthetic fuels for a net zero emissions world. Sustainability **9**(2) (2017). https://doi.org/10.3390/su9020306

[36] Schröder, A., Kunz, F., Meiss, J., Mendelevitch, R., von Hirschhausen, C.: Current and prospective costs of electricity generation until 2050. DIW Data Documentation 68, Berlin (2013). http://hdl.handle.net/10419/80348

[37] Global average levelised cost of hydrogen production by energy source and technology, 2019 and 2050 . International Energy Agency (2022). https://www.iea.org/data-and-statistics/charts/global-average-levelised-cost-of-hydrogen-production-by-energy-source-and-technology-2019-and-2050

[38] Palzer, A.: Optimierungsmodell REMod-D (2018). https://energiesysteme-zukunft.de/fileadmin/user_upload/Publikationen/PDFs/ESYS_Materialien_Optimierungsmodell_REMod-D.pdf3

[39] DTU International Energy Report 2014 . DTU (2014). https://backend.orbit.dtu.dk/ws/portalfiles/portal/102457047/DTU_INTL_ENERGY_REP_2014_WIND.pdf

[40] Härtel, P., Kristiansen, M., Korpas, M.: Assessing the impact of sampling and clustering techniques on offshore grid expansion planning. Energy Procedia **137** (2017). https://doi.org/10.1016/j.egypro.2017.10.342

[41] Schaber, K.: Integration of Variable Renewable Energies in the European power system: a model-based analysis of transmission grid extensions and energy sector coupling. PhD thesis, Technische Universität München, München (2014)

[42] Henning, H.-M., Palzer, A.: A comprehensive model for the german electricity and heat sector in a future energy system with a dominant contribution from renewable energy technologies—part i: Methodology. Renewable and Sustainable Energy Reviews **30**, 1003–1018 (2014). https://doi.org/10.1016/j.rser.2013.09.012

[43] Fraunhofer-Institut für Windenergie und Energiesystemtechnik (Fraunhofer IWES): Interaktion EE-Strom, Wärme und Verkehr (2015). https://www.iee.fraunhofer.de/de/projekte/suche/2015/interaktion_strom_waerme_verkehr.html

Acronyms

AEC Alkaline electrolysis cells

BECCS Bioenergy with carbon capture and storage

CHP Combined heat and power plant

DAC Direct air capture
DEA Danish Energy Agency

EPC Engineering, procurement and construction
ETC Energy Transitions Commission

FOM Fixed operation and maintenance costs
Fraunhofer ISE Fraunhofer Institute for Solar Energy Systems

ICE Internal combustion engine
IEA International Energy Agency
IRENA International Renewable Energy Agency

JRC Joint Research Center

MIP Mixed Integer Problem

NPV Net present value

PEM Polymer electrolyte membrane electrolysis
PV Solar photovoltaics

SCC Social cost of carbon
SMR Steam methane reforming
SOEC Solid oxide electrolysis cell

VOM Variable operation and maintenance costs

WACC Weighted average cost of capital
